# Tackling Complex Emergency Response Solutions Evaluation Problems in Sustainable Development by Fuzzy Group Decision Making Approaches with Considering Decision Hesitancy and Prioritization among Assessing Criteria

**DOI:** 10.3390/ijerph14101165

**Published:** 2017-10-02

**Authors:** Xiao-Wen Qi, Jun-Ling Zhang, Shu-Ping Zhao, Chang-Yong Liang

**Affiliations:** 1 School of Business Administration, Zhejiang University of Finance & Economics, Hangzhou 310018, China; 2 School of Economics and Management, Zhejiang Normal University, Jinhua 321004, China; zhangjunling@zjnu.cn; 3 School of Management, Hefei University of Technology, Hefei 230009, China; zhaoshuping1753@hfut.edu.cn (S.-P.Z.); cyliang@hfut.edu.cn (C.-Y.L.)

**Keywords:** emergency response solutions evaluation, multiple criteria group decision making, interval-valued dual hesitant fuzzy set, prioritized aggregation operators, fuzzy entropy

## Abstract

In order to be prepared against potential balance-breaking risks affecting economic development, more and more countries have recognized emergency response solutions evaluation (ERSE) as an indispensable activity in their governance of sustainable development. Traditional multiple criteria group decision making (MCGDM) approaches to ERSE have been facing simultaneous challenging characteristics of decision hesitancy and prioritization relations among assessing criteria, due to the complexity in practical ERSE problems. Therefore, aiming at the special type of ERSE problems that hold the two characteristics, we investigate effective MCGDM approaches by hiring interval-valued dual hesitant fuzzy set (IVDHFS) to comprehensively depict decision hesitancy. To exploit decision information embedded in prioritization relations among criteria, we firstly define an fuzzy entropy measure for IVDHFS so that its derivative decision models can avoid potential information distortion in models based on classic IVDHFS distance measures with subjective supplementing mechanism; further, based on defined entropy measure, we develop two fundamental prioritized operators for IVDHFS by extending Yager’s prioritized operators. Furthermore, on the strength of above methods, we construct two hesitant fuzzy MCGDM approaches to tackle complex scenarios with or without known weights for decision makers, respectively. Finally, case studies have been conducted to show effectiveness and practicality of our proposed approaches.

## 1. Introduction

Environmental problems that result from fast economic development planning, including chemical spills, explosions and underground water pollution, etc. are threatening our natural and socioeconomic systems [1,2]. Serious consequences have drawn extensive attention from local governments in the global village. To accommodate potential environmental risks, different countries have accumulatively established guidelines to instruct organizations in building emergency response solutions. Such as, the Organization for Economic Cooperation and Development (OECD) Guiding Principles for Chemical Accident Prevention [3], Hazardous Waste Operations and Emergency Response [4], Seveso III [5], etc. Aiming at bridging gaps between guidance and actual operations, academics have been working on effective emergency management methodologies and tools. For instance, Duan and He [6] designed an emergency response system to cope with potential safety incidents in chemical industry parks. Shao et al. [7] included response solutions as an imperative component in their whole-process environmental management framework. Most recently, Wan et al. [8] investigated the evaluation methodology for green port development and also emphasized the necessity of response solutions in case of extreme negative effects caused by climate change. As can be seen, getting prepared with emergency response solutions is the intrinsic part of effective ways to address potential environmental risks in the course of achieving sustainable development. 

Although governments have continuously constructed a set of contingency solutions to known disastrous events, economic development programs often break the balance by introducing new threats to public health, such as the epidemic emergency event due to imported Zika virus in the YiWu International Trading Community of China [9], risks by technology-driven industries (such as nanotextiles, smart-textiles and chemical medicines) [10,11], and risks introduced by disreputable aluminum ore mining industries in developing countries. Therefore, in order to get well prepared to respond to potential risks, governments at different levels are behooved to establish decision support systems for emergency management [12,13], and to adopt decision-making procedures for risk assessment and emergency response solutions evaluation (ERSE) [1,13]. ERSE is thus observed as an indispensable activity in governance for sustainable development. 

For the reason that ERSE activities generally involve a group of stakeholders to comprehensively consider response solutions under a set of criteria, ERSE is normally conceptualized as a type of multi-criteria group decision making (MCGDM) problem [1,9,14,15,16,17], in which decision preferences are often uncertain due to problem complexity. To effectively accommodate the uncertain multi-criteria decision making problems, many efforts have been expended [18], such as the extended ELECTRE-III under interval-valued intuitionistic fuzzy environment [19], complex proportional assessment (COPRAS) approaches [20,21], the additive ratio assessment (ARAS) methods [22,23], Multiobjective Optimisation by Ratio Analysis Plus Full Multiplicative Form (MULTIMOORA) [24,25,26], etc. Regarding ERSE problems of uncertainty, only limited literature can be found currently, such as, Ju et al. [15] who applied an analytical hierarchy process (AHP) to evaluate the capacity of emergency response solutions based on linguistic assessments. Ju and Wang [14] who adopted Dempster-Shafer theory and the AHP method to investigate problems with incomplete and uncertain information. Moreover, Ju and Yang [16] have employed a trapezoid fuzzy linguistic set to effectively elicit fuzzy uncertain opinions of decision makers. Wang et al. [17] introduced prospect theory under interval uncertain environments to simulate dynamic reference points of decision makers’ psychological behaviors. Unfortunately, when asking decision makers to give membership degrees or non-membership degrees to a fuzzy set, they usually behave hesitantly among possible values, so to tackle this complicated decision-making situations with decision hesitancy, Torra and Narukawa [27] and Torra [28] suggested hesitant fuzzy set (HFS) to allow membership degrees of an element to be a set of possible crisp values. Because of its merits in expressing hesitant information, HFS has been widely studied for more complex situations, including the dual hesitant fuzzy set (DHFS) [29] that contains both possible membership degrees and non-membership degrees, interval-valued dual hesitant fuzzy set (IVDHFS) [30,31] that more practically allows possible membership or non-membership degrees to be interval values. Most recently, aiming at the complex ERSE problems where decision hesitancy exists and weighting information for both criteria and decision makers cannot be determined in advance, Zhang et al. [9] developed two approaches that utilized IVDHFS to elicit hesitant fuzzy evaluations. However, in their approaches, the distance measure of IVDHFS that was adopted to derive weighting information obeyed a classic complementing method [32,33], i.e., optimistic or pessimistic mechanism of filling the unmatched membership degree set or non-membership degree set in any two compared interval-valued dual hesitant fuzzy elements, but apparently, the value-filling mechanism will inevitably cause some information distortion in contrast with the original preference information. Fortunately, programming models based on fuzzy entropy measures [34,35] have been shown to be another effective way to derive unknown weighting information, and more importantly, these entropy-based models only depend on information originally given by decision makers, thus they can avoid the potential information distortion from subjective supplementation. Examples are models based on interval-valued intuitionistic fuzzy entropy [36,37] and cross-entropy [38], cross-entropy for interval neutrosophic set [39], hesitant fuzzy entropy and cross-entropy [40], dual hesitant fuzzy cross-entropy [41], etc. However, up to now, scarcely any related research has been carried out on entropy measures for IVDHFS. Therefore, in this paper, to tackle complex ERSE problems more effectively and objectively, we follow the idea of classic fuzzy entropy [34,35] to develop an entropy measure for IVDHFS in the first place. 

From another perspective, due to evolving emergency event scenarios, ERSE usually has event-dependent characteristics pertaining to decision making processes, and some efforts have been carried out on various issues, such as Xie et al. [42] who proposed an multi-round agile Delphi-based group decision-making method in which decision makers’ preference adjustments on events were dynamically mapped to changes of their weights. By incorporating cellular automata, Cao et al. [43] developed an integrated emergency response evaluation approach to select optimal evacuation routes for toxic gas release accidents. Shi et al. [44] constructed a treatment and disposal response plan repository for chemical pollution accidents by employing an event-tree method to simulate dynamic processing changes, then utilized an AHP-based MCGDM method to evaluate response plans. In order to select emergency treatment technologies for chemical contingency spills, Liu et al. [45] presented a fuzzy grey relational analysis-based multiple criteria decision making method with support of a dynamic fuzzy aggregation average operator that considers effects of time periods. Wang et al. [17] noted the dynamic effects of decision makers’ psychological behaviors on assessments, and thus employed prospect theory to establish event-dependent reference points in their TOPSIS-based approach. However, there is still a lack of related literature on ERSE that has noted event-dependent prioritization phenomena among assessing criteria, especially under complex uncertain scenarios. Although AHP-based methods are applied to differentiate the relative importance of assessment criteria, their reciprocal comparisons generally expect precisely consistent judgements from decision makers otherwise multiple rounds of adjustments will be needed to improve the judgement consistency, which behooves the existence of differentiating procedures rather than complicated and time-consuming ones, while in practice, if confronted with emergency decision scenarios, decision makers generally are capable of approximating fairly the accurate prioritization relations among assessing criteria, based on their expertise and experience. For example, suppose four criteria, i.e., response efficiency (*C*_1_), environmental impact (*C*_2_), social impact (*C*_3_) and cost (*C*_4_), are to be considered to select response solutions for chemical spill events. If the event spot A_1_ was in a desert area with scarce residences but freeways with regular traffic, decision makers would naturally deduce the prioritization relation among the four criteria as: *C*_1_ ≻ *C*_4_ ≻ *C*_3_ ≻ *C*_2_; while if the spot A_1_ was located nearby a residential district, decision makers would derive a different prioritization *C*_1_ ≻ *C*_3_ ≻ *C*_2_ ≻ *C*_4_. In actuality, prioritization phenomena generally exist in problem-oriented multiple criteria decision making, even in everyday life. Taking the example of sedan purchase decision making, most people would consider: safety ≻ (regular maintenance and price) ≻ comfort, while most hedonistiic people would perceive: safety ≻ comfort ≻ (regular maintenance and price). Obviously, effective MCGDM approaches should be developed to accommodate practical ERSE problems with prioritization relations among assessing criteria. 

Fortuitously, from the perspective of information fusion, Yager [46,47] proposed the prioritized average (PA) operator and the prioritized ordered weighted average (POWA) operator, which provide an effective way to reflect both assessments under criteria and prioritization relations among those criteria. Since then, prioritized operators have been developed in-depth to accommodate complicate uncertain decision environments, such as those extended prioritized operators under intuitionistic fuzzy environments [48,49,50,51], and those under multi-granular uncertain linguistic environments [52]. The prioritized average operator also has been integrated into the classical PROMETHEE method for handling prioritized multi-criteria decision making [53]. As for more complex decision making scenarios where decision hesitancy exists [27,28], Wei [54] developed some extended prioritized operators. Wu et al. [55] further investigated decision making approaches based on their proposed generalized hesitant fuzzy linguistic prioritized operators. Jin et al. [56] extended this work to put forward Einstein operations-based prioritized operators to accommodate interval-valued hesitant fuzzy decision information, based on which they further constructed decision making approaches and applied to resolve a talent recruitment problem. With respect to the equal importance of non-membership degrees in depicting decision hesitancy [29], only Ren and Wei [57] have investigated a multiple criteria decision-making method with prioritization relationship based on a dice similarity, but their method was restricted to single-person decision-making with crisp membership and crisp non-membership degrees. In actuality, when confronted with complex decision problems, decision makers are often inclined to or only capable of expressing membership or non-membership degrees by use of interval values [30,31]. Apparently, there is a practical and significant need to investigate prioritized average aggregation operators and their derivative effective multiple criteria decision making approaches under interval-valued dual hesitant fuzzy environment conditions. 

Therefore, in this paper, focusing on the special type of practical ERSE problems where there exist phenomena of prioritization among assessment criteria and there exist hesitant decision preferences, we firstly employ IVDHFS to elicit hesitant fuzzy assessments from decision makers more effectively and comprehensively. To rationally exploit decision information embedded in prioritization relations among assessing criteria, we develop two fundamental prioritized average operators for IVDHFS, i.e., the interval-valued dual hesitant fuzzy prioritized average (IVDHFPWA) operator and the interval-valued dual hesitant fuzzy prioritized ordered weighted average (IVDHFPOWA) operator. Furthermore, to objectively derive associated weighting vectors in the proposed IVDHFPWA and IVDHFPOWA operators, we adapt their averaging mechanism to rely on our newly defined entropy measure for IVDHFS. Moreover, according to specific complicated ERSE settings whether weights for decision makers can be obtained in advance or not, we propose two MCGDM approaches, respectively.

The rest of this paper is organized as follows: in Section 2, we explain the special type of ERSE problems with prioritization relations among assessment criteria, then formulate them concisely. In Section 3, we define a fuzzy entropy measure for IVDHFS. Then in Section 4, the IVDHFPWA operator and the IVDHFPOWA operator are proposed, and their desirable properties are also analyzed. Subsequently in Section 5, on the strength of the above developed tools, we construct two MCGDM approaches to tackle complex ERSE problems while simultaneously considering decision hesitancy and prioritization relations among assessment criteria. Section 6 provides some experimental studies to verify the effectiveness and practicality of our approaches. Finally, conclusions are drawn as well as future research directions proposed in Section 7. 

## 2. ERSE with Prioritization among Assessing Criteria

Generally, an emergency response solution is a set of procedures to be implemented in case of certain catastrophic event situation, which involves risk analysis, communication, intervention actions, operational supports, logistics supports and whatever is necessary to reduce harmful impacts [1,13,58]. 

Due to the fact that enforcement of emergency response solutions intrinsically involves multiple participants, from the relatively macroscopic point of view, it has been suggested by academics that emergency responses be implemented based on collaborative organization paradigms so as to achieve relief objectives [59,60,61,62]. In view of that multi-organizational nature of large-scale disaster response has been a challenging collaborative information system development, Maldonado et al. [59] investigated a multi-level and multi-organizational information system coordination framework, verified advantageous resource transferral along different organization levels and changeable outcomes of authority coercion in their framework. Focusing on the structural complexity issue in governance networks, Koliba at al. [60] identified explicit and implicit standards of collaborative accountability, such as written agreements, decision making procedures, reciprocity, and durability of relationships. Kuo et al. [61] studied lessons from collaborative disaster management in Taiwan’s local governments, and derived three crucial factors to effective collaborative disaster response, including trust, information sharing and accountability. Noran [62] argued that emergency response services from a heterogeneous set that often underperforms due to a lack of appropriate interoperation and collaboration, then designed an collaborative response paradigm that was enabled by discipline integration of interoperability, collaborative networks and enterprise architecture. By use of network analysis [63], Guo and Kapucu [64] examined China’s emergency response governance, which is characterized with a hierarchical organizational structure and centralized control, and its inherent obstacles to effective coordination and collaboration. 

While from a more microscopic perspective of emergency management, effective emergency response heavily relies on planned emergency response solutions to various identified events [65]. As a result, local governments have laid down regulations or laws to construct emergency response solutions against potential risks that frequently come along with economic exploitation programs, especially in those countries with high population density and investment density. For example, although the developing country of China actually confronts organizational-structural issues in collaborative governance on emergency response, the Chinese central government still enacted instructional regulations about nation-wide emergency response plans in 2011 [66] Since then, local governments in China have included ERSE as one of the indispensable tasks in their emergency management departments. 

Due to the complexity of ERSE problems and their requirements of decision-making in settings with a nexus of stakeholders, MCGDM methodologies have been widely employed to fulfil the evaluation tasks [9]. Representatively, Ju and Wang [14] and Ju et al. [1] have been working on effective MCGDM approaches to evaluate emergency response solutions on the premise of saving lives or property in the first place. They set up an assessing criteria index from the perspective of functional division in emergency response solutions, but they neglected the factors of social and environmental influences during or after events. For example, from the tactical point of view in coping with chemical pollution events, Shi et al. [44] developed the AHP-based MCGDM method for evaluation and selection of the most desirable solutions to emergency accidents, in which they explicitly included the factors of economic cost, social influence and environmental influence in their criteria system, although the criteria system lacked systematical consideration of emergency response activities from a lifecycle perspective. In actuality, emergency management practices [67] have emphasized that effective and efficient response solutions should be well prepared coherently, including early warning, event identifying and classification, information acquisition, supportive functioning division, recovery, analysis on social impact and environmental impact, etc. That is, emergency response solution response evaluation should consider desirable indicators to cover three stages: pre-activity, during-activity and after-activity [68,69]. Therefore, inspired by the above-mentioned work, we here derive the following assessing criteria system according to lifecycle model, as shown in Table 1. 

More importantly, due to the fact that ERSE tasks are strongly event-dependent activities, distinct event scenarios usually cause the phenomena of prioritization among assessment criteria. Taking example of the aniline leak accident in the Zhang River Basin of China in 2012, to determine the most appropriate emergency response solutions, Shi at al. [44] set up a criteria system for evaluating alternative solutions from four aspects, including technical performance, economic cost, environmental impact and social impact. The leak accident happened near Lucheng City and posed a potential threat to the Yuecheng Reservoir drinking water source for the city residents. Because saving lives is always first prioroty, the criterion of technical performance should be endowed with the highest priority level, and the criterion of economic cost should be left with the least priority level correspondingly. For the reason that the accident will probably contaminate water supplies and thus cause public panic along with cascading emergency events, the criterion of social impact should be the adjoining one that is inferior to the criterion of technical performance and is superior to the criterion of environmental impact. In sum, the prioritization relationship among the above four criteria can be deduced as: technical performance ≻ social impact ≻ environmental impact ≻ economic cost, which was verified by the analytical hierarchy process method in [44]. However, this prioritization relationship will change in other event scenarios, for instance, if the aniline leak accident under discussion was located near the water resources in a natural landscape area but far from any city or community, then we expect that decision makers will naturally deduce the prioritization: environmental impact ≻ social impact. In practice, emergency departments generally are prompted to follow guidelines stipulated in special laws or regulations of different countries [70,71], thereby constructing more comprehensive criteria system for distinct types of response solutions, such as the activity phase-oriented assessing criteria shown in Table 1. Still, when applying the criteria system to practical ERSE, we usually also have to rationally infer prioritization relations among criteria based on the characteristics of various emergency events, such as the examples shown in Table 2. As seen from the above analysis, prioritization relations among criteria usually exist in ERSE activities and they vary according to the specific event scenario. 

Therefore, in this paper, we focus on investigating this special type of practical ERSE problems with prioritization relations among assessing criteria, which can be formulated concisely as follows.

Given an emergency event, to which there are a set of alternative response solutions: $X=\{x_{1},x_{2},\ldots ,x_{n}\}$. Let $C=\{C_{1},C_{2},\ldots ,C_{m}\}$ be the assessing criteria which fall into one of the prioritization levels which signified as $H=\{H_{1},H_{2},\ldots ,H_{m}\}$. We use $C_{j}^{u}$ to denote that the *j*-th (j=1,…,m) criterion is assigned with the *u*-th (u=1,…,m) prioritization level $H_{u}$. Because the ERSE activities are intrinsically complicate and generally single decision maker only cannot assess response solutions from hybrid perspectives, thus a group of decision makers $E=\{E_{1},E_{2},\ldots ,E_{t}\}$ should be organized to consider all the response solutions $x_{i}$ (i=1,…,n) under each criterion $C_{j}$ (j=1,…,m). Subsequently, after decision makers depict their preferences with suitable expression tools, we collect and construct their decision matrices $R^{k}={\left(r_{ij}\right)}_{m\times n}$ (*k* = 1, …, *t*), which normally take the format as shown in Table 3. Then, based on the decision information in Table 3, effective multiple criteria group decision making approaches should be developed to help city emergency management departments determine the most appropriate response solution(s). 

Usually, due to complexity in ERSE problem definition and the limited cognition of human beings, decision makers are not straightforwardly capable of expressing their assessments in $R^{k}={\left(r_{ij}\right)}_{n\times m}$ with crisp numbers, especially when decision makers are uncertain and hesitate about their assessments. The literature thus encourages introduction of comprehensive expression tools to help decision makers effectively elicit their actual preferences, such as linguistic variables [1,16], intuitionistic fuzzy set [38,75], etc. 

Nevertheless, regarding ill-structured complicated ERSE problems, decision makers can only achieve approximate judgements and usually will hesitate about possible membership degrees [28] or non-membership degrees [29] of their fuzzy opinions. To accommodate the uncertainty of hesitancy, Farhadinia [30] and Ju et al. [31] introduced the conceot of interval-valued dual hesitant fuzzy set (IVDHFS), which attains more practicality and flexibility in comparison to the conventional hesitant fuzzy set (HFS) [28] and dual hesitant fuzzy set (DHFS) [29]. However, existing MCGDM [18,76] based on IVDHFS were mostly derived from the supplementing distance measure [32,33] that will cause distortion to some extent due to its artificial value-filling mechanism. Therefore, inspired by the classic fuzzy entropy measure [34,35,37,77,78,79,80] and its derivative methodologies [37,38], we develop an entropy-based distance measure for IVDHFS in the following sections to avoid the limitations of methodologies based on the supplementing distance measure. Furthermore, by use of the IVDHFS distance measure, we propose two aggregation operators to utilize decision information embedded in the prioritization relationships objectively. Afterwards, on the basis of the developed operators, we construct two effective MCGDM approaches to resolve the type of complex ERSE problems with decision hesitancy and prioritization relations among assessment criteria. 

## 3. An Entropy Measure for IVDHFS

### 3.1. Basic Notions of IVDHFS

Torra and Narukawa [27] and Torra [28] proposed a hesitant fuzzy set (HFS) to manage those situations where several values are possible for defining a membership function of a given fuzzy concept, while Zhu et al. [29] pointed out that non-membership degrees take on equal importance s membership degrees when depicting the hesitancy of decision makers, they thus extended HFS to the dual hesitant fuzzy set (DHFS). Further, by noticing the fact that decision makers are often inclined to assign interval numbers to membership degrees or non-membership degrees, Farhadinia [30] and Ju et al. [31] proposed the IVDHFS to attain more practicality and flexibility. In the following, we firstly brief basic notions of IVDHFS.

**Definition** **1** **[30,31].** *Let* X *be a fixed set, then an interval-valued dual hesitant fuzzy set (IVDHFS) on* X *is defined as:*

(1) $$\tilde{A}=\left\{\langle x,\tilde{h}(x),\tilde{g}(x)\rangle |x\in X\right\},$$

*where* $\tilde{h}(x)=\cup_{\left[\mu^{L},\mu^{U}]\in \tilde{h}(x\right)}\left\{\tilde{\mu }\right\}=\cup_{\left[\mu^{L},\mu^{U}]\in \tilde{h}(x\right)}\left\{[\mu^{L},\mu^{U}]\right\}$*,* $\tilde{g}(x)=\cup_{\left[\nu^{L},\nu^{U}]\in \tilde{g}(x\right)}\left\{\tilde{\nu }\right\}=\cup_{\left[\nu^{L},\nu^{U}]\in \tilde{g}(x\right)}\left\{[\nu^{L},\nu^{U}]\right\}$ *are two sets of several interval values in [0, 1], denoting possible membership degrees and possible non-membership degrees of element* x∈X *to* $\tilde{A}$*, respectively. And following conditions be satisfied:* $\tilde{\mu },\tilde{\nu }\in [0,1]$*,* $0\leq {\left(\mu^{U}\right)}^{+}+{\left(\nu^{U}\right)}^{+}\leq 1$*, where* ${\left(\mu^{U}\right)}^{+}\in {\tilde{h}}^{+}(x)=\cup_{\left[\mu^{L},\mu^{U}]\in \tilde{h}(x\right)}\max\{\mu^{U}\}$ *and* ${\left(\nu^{U}\right)}^{+}\in {\tilde{g}}^{+}(x)=$ $\cup_{\left[\nu^{L},\nu^{U}]\in \tilde{g}(x\right)}\max\{\nu^{U}\}$*for all* x∈X*.*

*Here* $\tilde{\alpha }=(\tilde{h},\tilde{g})$ *is called an interval-valued dual hesitant fuzzy element (IVDHFE), then we use* $\tilde{A}$ *to denote the set of all interval-valued dual hesitant fuzzy elements (IVDHFEs).*

**Definition** **2** **[31].** *Considering any three IVDHFEs:* $\tilde{\alpha }=(\tilde{h},\tilde{g})$*,* ${\tilde{\alpha }}_{1}=({\tilde{h}}_{1},{\tilde{g}}_{1})$*, and* ${\tilde{\alpha }}_{2}=({\tilde{h}}_{2},{\tilde{g}}_{2})$*, let* λ>0*, then some basic operations on them are defined as:*

*(1)*  ${\tilde{\alpha }}^{\lambda }=\cup_{[\mu^{L},\mu^{U}]\in \tilde{h},[\nu^{L},\nu^{U}]\in \tilde{g}}\left\{\{[{\left(\mu^{L}\right)}^{\lambda },{\left(\mu^{U}\right)}^{\lambda }]\},\{[1-{\left(1-\nu^{L}\right)}^{\lambda },1-{\left(1-\nu^{U}\right)}^{\lambda }]\}\right\}$*;*
*(2)*  $\lambda \tilde{\alpha }=\cup_{[\mu^{L},\mu^{U}]\in \tilde{h},[\nu^{L},\nu^{U}]\in \tilde{g}}\left\{\{[1-{\left(1-\mu^{L}\right)}^{\lambda },1-{\left(1-\mu^{U}\right)}^{\lambda }]\},\{[{\left(\nu^{L}\right)}^{\lambda },{\left(\nu^{U}\right)}^{\lambda }]\}\right\}$*;*
*(3)*  ${\tilde{\alpha }}_{1}⊕{\tilde{\alpha }}_{2}=\cup_{[\mu_{1}^{L},\mu_{1}^{U}]\in {\tilde{h}}_{1},[\mu_{2}^{L},\mu_{2}^{U}]\in {\tilde{h}}_{2},[\nu_{1}^{L},\nu_{1}^{U}]\in {\tilde{g}}_{1},[\nu_{2}^{L},\nu_{2}^{U}]\in {\tilde{g}}_{2}}$
  $\left\{\{[\mu_{1}^{L}+\mu_{2}^{L}-\mu_{1}^{L}\mu_{2}^{L},\mu_{1}^{U}+\mu_{2}^{U}-\mu_{1}^{U}\mu_{2}^{U}]\},\{[\nu_{1}^{L}\nu_{2}^{L},\nu_{1}^{U}\nu_{2}^{U}]\}\right\}$*;*
*(4)*  ${\tilde{\alpha }}_{1}⊗{\tilde{\alpha }}_{2}=\cup_{[\mu_{1}^{L},\mu_{1}^{U}]\in {\tilde{h}}_{1},[\mu_{2}^{L},\mu_{2}^{U}]\in {\tilde{h}}_{2},[\nu_{1}^{L},\nu_{1}^{U}]\in {\tilde{g}}_{1},[\nu_{2}^{L},\nu_{2}^{U}]\in {\tilde{g}}_{2}}$
  $\left\{\{[\mu_{1}^{L}\mu_{2}^{L},\mu_{1}^{U}\mu_{2}^{U}]\},\{[\nu_{1}^{L}+\nu_{2}^{L}-\nu_{1}^{L}\nu_{2}^{L},\nu_{1}^{U}+\nu_{2}^{U}-\nu_{1}^{U}\nu_{2}^{U}]\}\right\}$*.*

**Definition** **3** **[31].** *Let* ${\tilde{\alpha }}_{1}=({\tilde{h}}_{1},{\tilde{g}}_{1})$ *and* ${\tilde{\alpha }}_{2}=({\tilde{h}}_{2},{\tilde{g}}_{2})$ *be any two IVDHFEs,* λ>0*, then the basic operational rules on them are defined as:*

*(1)*  ${\tilde{\alpha }}_{1}⊕{\tilde{\alpha }}_{2}={\tilde{\alpha }}_{2}⊕{\tilde{\alpha }}_{1}$*;*
*(2)*  ${\tilde{\alpha }}_{1}⊗{\tilde{\alpha }}_{2}={\tilde{\alpha }}_{2}⊗{\tilde{\alpha }}_{1}$*;*
*(3)*  $\lambda ({\tilde{\alpha }}_{1}⊕{\tilde{\alpha }}_{2})=\lambda {\tilde{\alpha }}_{1}⊕\lambda {\tilde{\alpha }}_{2}$*;*
*(4)*  ${\tilde{\alpha }}_{1}^{\lambda }⊗{\tilde{\alpha }}_{2}^{\lambda }={\left({\tilde{\alpha }}_{2}⊗{\tilde{\alpha }}_{1}\right)}^{\lambda }$*.*

*In addition, to compare any two IVDHFEs, Ju, Liu and Yang* [31] *also introduced a score function* $s(\tilde{\alpha })$ *to compute scores of a given* *IVDHFE* $\tilde{\alpha }$ *and an accuracy function* $v(\tilde{\alpha })$ *to evaluate accuracy degrees of* $\tilde{\alpha }$*, where*

(2) $$s(\tilde{\alpha })=\frac{1}{2}\left(\frac{1}{l(\tilde{h})}\sum_{[\mu^{L},\mu^{U}]\in \tilde{h}}\mu^{L}-\frac{1}{l(\tilde{g})}\sum_{[\nu^{L},\nu^{U}]\in \tilde{g}}\nu^{L}+\frac{1}{l(\tilde{h})}\sum_{[\mu^{L},\mu^{U}]\in \tilde{h}}\mu^{U}-\frac{1}{l(\tilde{g})}\sum_{[\nu^{L},\nu^{U}]\in \tilde{g}}\nu^{U}\right),$$

(3) $$v(\tilde{\alpha })=\frac{1}{2}\left(\frac{1}{l(\tilde{h})}\sum_{[\mu^{L},\mu^{U}]\in \tilde{h}}\mu^{L}+\frac{1}{l(\tilde{g})}\sum_{[\nu^{L},\nu^{U}]\in \tilde{g}}\nu^{L}+\frac{1}{l(\tilde{h})}\sum_{[\mu^{L},\mu^{U}]\in \tilde{h}}\mu^{U}+\frac{1}{l(\tilde{g})}\sum_{[\nu^{L},\nu^{U}]\in \tilde{g}}\nu^{U}\right).$$

*Here* $l(\tilde{h})$ *and* $l(\tilde{g})$ *are the numbers of interval values in* $\tilde{h}$ *and* $\tilde{g}$*, respectively. The larger the score* $S(\tilde{\alpha })$*, the larger the accuracy* $v(\tilde{\alpha })$*, the greater the IVDHFE* $\tilde{\alpha }$*. Then the ordering relation between any two IVDHFEs* ${\tilde{\alpha }}_{1}=({\tilde{h}}_{1},{\tilde{g}}_{1})$ *and* ${\tilde{\alpha }}_{2}=({\tilde{h}}_{2},{\tilde{g}}_{2})$ *can be determined according to the following rules:*

*If* $s({\tilde{\alpha }}_{1})<s({\tilde{\alpha }}_{2})$*, then* ${\tilde{\alpha }}_{1}<{\tilde{\alpha }}_{2}$*.*

*If* $s({\tilde{\alpha }}_{1})=s({\tilde{\alpha }}_{2})$*, then*

*(1)*  *If* $v({\tilde{\alpha }}_{1})=v({\tilde{\alpha }}_{2})$*, then* ${\tilde{\alpha }}_{1}={\tilde{\alpha }}_{2}$*;*
*(2)*  *If* $v({\tilde{\alpha }}_{1})<v({\tilde{\alpha }}_{2})$*, then* ${\tilde{\alpha }}_{1}<{\tilde{\alpha }}_{2}$*.*

### 3.2. An Entropy Measure for IVDHFS

Regarding the MCGDM problems of high uncertainty, weighting information often cannot be determined in advance, so different approaches have thus been developed to derive the unknown weighting information. Representatively: (i) maximum deviation programming methodologies for obtaining unknown criteria weights, such as those models for intuitionistic fuzzy decision environment [81], for hesitant fuzzy decision environment [40] and for interval-valued dual hesitant fuzzy environment [9]; (ii) similarity-based methodologies for deriving unknown weights of decision makers, such as the compatibility maximizing model based on similarity of one decision matrix to others [9]. 

As can be seen, the above methodologies generally depend on appropriate distance measures for specific forms of decision information. However, due to the fact that the length of membership degrees or non-membership degrees of one hesitant fuzzy element generally does not match the length of the other compared element, preceding efforts in the literature mostly adopted the classic supplementing distance measures [32,33], which extends the shorter sets of membership degrees or non-membership degrees by adding values till the lengths match. As a result, although this supplementing mechanism reflects two attitudes of decision makers, subjectively adding values will inevitably cause information distortion to some extent. 

Meanwhile, the methods based on fuzzy entropy [34,35] have provided us another effective ways to objectively utilize assessments in determining unknown weighting information. Emblematically, Ye [36] and Ye [37] developed an entropy-based model to obtain criteria weights under intuitionistic fuzzy environments; Qi et al. [38] proposed an generalized interval-valued intuitionistic fuzzy cross-entropy measure, based on which two programming model were developed to obtain unknown criteria weights and unknown expert weights; Xu and Xia [40] investigated entropy measure and cross-entropy measure for HFS, then designed a straightforward entropy-based criteria weighting method. With respect to dual hesitant fuzzy decision making scenarios, only recently, Zhao and Xu [82] studied some entropy measures and discussed their properties, Ye [41] defined a dual hesitant fuzzy cross-entropy measure and utilized it to construct an ideal solution-based decision making approach. Nevertheless, to the best of our knowledge, there is scarcely any effort that has been carried out to investigate entropy measures for IVDHFS and derivative MCGDM approaches. Therefore, we here introduce an entropy measure for IVDHFS and discuss its properties.

**Definition** **4.** *Given an interval-valued dual hesitant fuzzy element (IVDHFE)* $\tilde{\alpha }$*, the following*

(4) $$e(\tilde{\alpha })=1-d\left(\tilde{\alpha },\left(\left\{\left[\frac{1}{2},\frac{1}{2}\right]\right\},\left\{\left[\frac{1}{2},\frac{1}{2}\right]\right\}\right)\right),$$

*is an entropy for IVDHFEs, where distance* d *is calculated by the normalized Hamming distance according to*

(5) $$d\left(\tilde{\alpha },\left(\left\{\left[\frac{1}{2},\frac{1}{2}\right]\right\},\left\{\left[\frac{1}{2},\frac{1}{2}\right]\right\}\right)\right)=\frac{1}{2l_{1}}\sum_{j=1}^{l_{1}}\left(\left|\mu_{j}^{L}-\frac{1}{2}\right|+\left|\mu_{j}^{U}-\frac{1}{2}\right|\right)+\frac{1}{2l_{2}}\sum_{j=1}^{l_{2}}\left(\left|\nu_{j}^{L}-\frac{1}{2}\right|+\left|\nu_{j}^{U}-\frac{1}{2}\right|\right).$$

Regarding the above entropy measure defined in Definition 4, we have the following theorem to verify it meets the axiomatic requirements of a fuzzy entropy [40].

**Theorem** **1.** *The entropy* $e(\tilde{\alpha })$ *on* $\tilde{\alpha }$ *satisfies the following requirements:*

*(1)*  $0\leq e(\tilde{\alpha })\leq 1$*;*
*(2)*  $e(\tilde{\alpha })=0$*, if and only if* $\tilde{\alpha }=\left(\left\{[1,1]\},\{[0,0]\right\}\right)$ *or* $\tilde{\alpha }=\left(\left\{[0,0]\},\{[1,1]\right\}\right)$*;*
*(3)*  $e(\tilde{\alpha })=1$*, if and only if* $\tilde{\alpha }=\left(\left\{\left[\frac{1}{2},\frac{1}{2}\right]\right\},\left\{\left[\frac{1}{2},\frac{1}{2}\right]\right\}\right)$*;*
*(4)*  $e(\tilde{\alpha })=e({\tilde{\alpha }}^{c})$*;*
*(5)*  $e({\tilde{\alpha }}_{1})\leq e({\tilde{\alpha }}_{2})$ *if* ${\tilde{\alpha }}_{1}$ *is less fuzzy than* ${\tilde{\alpha }}_{2}$*.*

All the properties listed in Theorem 1 are straightforward, thus their proofs are omitted here. In addition, it is worth mentioning that the proposed entropy measure does not need any artificially added values to supplement IVDHFEs, thus its derivative weighting approaches are capable of avoiding the potential information distortion as caused by the classic supplementing methods in [32,33].

**Example** **1.** *Let* ${\tilde{\alpha }}_{1}=\left(\left\{[0.1,0.2]\},\{[0.7,0.8]\right\}\right)$*,* ${\tilde{\alpha }}_{2}=\left(\left\{[0.4,0.5],[0.5,0.6]\},\{[0.2,0.3]\right\}\right)$*, then according to Definition 4, we have* $e({\tilde{\alpha }}_{1})$ *= 0.4,* $e({\tilde{\alpha }}_{2})$ *= 0.7.*

## 4. Interval-Valued Dual Hesitant Fuzzy Prioritized Average Operators

To facilitate multiple criteria decision making based on interval-valued dual hesitant fuzzy information, Ju et al. [31] have developed some basic aggregation operators for developing effective approaches. Among those operators, one of the most adopted is the interval-valued dual hesitant fuzzy weighted average (IVDHFWA) operator as shown in the following Definition 5.

**Definition** **5** **[31].** *For a collection of IVDHFEs:* ${\tilde{\alpha }}_{j}(j=1,2,\ldots ,n)$*, an interval-valued dual hesitant fuzzy weighted average (IVDHFWA) operator is a mapping of* $S^{n}\to S$*, such that*

(6) $$IVDHFWA({\tilde{\alpha }}_{1},{\tilde{\alpha }}_{2},\ldots ,{\tilde{\alpha }}_{n})=\overset{n}{\underset{j=1}{⊕}}(\omega_{j}{\tilde{\alpha }}_{j})\;=\cup_{[\mu_{j}^{L},\mu_{j}^{U}]\in {\tilde{h}}_{j},[\nu_{j}^{L},\nu_{j}^{U}]\in {\tilde{g}}_{j}}\left\{\left\{\left[1-\prod_{j=1}^{n}{\left(1-\mu_{j}^{L}\right)}^{\omega_{j}},1-\prod_{j=1}^{n}{\left(1-\mu_{j}^{U}\right)}^{\omega_{j}}\right]\right\},\left\{\left[\prod_{j=1}^{n}{\left(\nu_{j}^{L}\right)}^{\omega_{j}},\prod_{j=1}^{n}{\left(\nu_{j}^{U}\right)}^{\omega_{j}}\right]\right\}\right\}$$

*where* $\omega ={\left(\omega_{1},\omega_{2},\ldots ,\omega_{n}\right)}^{T}$ *is weights for* ${\tilde{\alpha }}_{j}(j=1,2,\ldots ,n)$*, with* $\omega_{j}\in [0,\;1]$ *and* $\sum_{j=1}^{n}\omega_{j}=1$*.*

However, the IVDHFWA operator and other elementary operators cannot accommodate multiple criteria decision making problems where there exists prioritization among assessment criteria. Actually, Yager [46] already noticed the real-world prioritization phenomena among assessing criteria, and thus developed the prioritized average (PA) operator [46] and the prioritized ordered weighted average (POWA) operator [47], as shown in the following Definitions 6 and 7.

**Definition** **6** **[46].** *Let* $C=\{c_{1},c_{2},\ldots ,c_{n}\}$ *be a collection of criteria and that there is* a priori*tization among the criteria expressed by the linear ordering* $c_{1}≻c_{2}≻\ldots ≻c_{n}$*, indicating criteria* $c_{j}$ *has a higher priority than* $c_{k}$ *if* j<k*. The value* $c_{j}(x)$ *is the performance of any alternative under any criteria* $c_{j}$*, and satisfies* $c_{j}\in [0,1]$*, if*

(7) $$PA(c_{j}(x))=\sum_{j=1}^{n}\omega_{j}c_{j}(x),$$

*where* $\omega_{j}=\frac{T_{j}}{\sum_{j=1}^{n}T_{j}}$*,* $T_{1}=1$ *and* $T_{j}=\prod_{k=1}^{j-1}c_{k}(x)$*, then PA is called the prioritized weighted average (PA) operator.*

**Definition** **7** **[47].** *Let* $C=\{c_{1},c_{2},\ldots ,c_{n}\}$ *be a collection of criteria and that there is* a priori*tization among the criteria expressed by the linear ordering* $c_{1}≻c_{2}≻\ldots ≻c_{n}$*, indicating criteria* $c_{j}$ *has a higher priority than* $c_{k}$ *if* j<k*. The value* $c_{j}(x)$ *is the performance of any alternative under any criteria* $c_{j}$*, and satisfies* $c_{j}\in [0,1]$*, we have the prioritized ordered weighted average (POWA) operator as following*

(8) $$POWA(c_{j}(x))=\sum_{j=1}^{n}\omega_{j}c_{ind(j)}(x),$$

(9) $$\omega_{j}=Q\left(\frac{\sum_{k=1}^{j}T_{ind(k)}}{\sum_{j=1}^{n}T_{j}}\right)-Q\left(\frac{\sum_{k=1}^{j-1}T_{ind(k)}}{\sum_{j=1}^{n}T_{j}}\right),j=1,2,\ldots ,n,$$

*in which* ind(⋅) *is a permutation function such that* ind(j−1)≥ind(j)*,* $T_{1}=1$*,* $T_{ind(0)}=0$ *and* $T_{j}=\prod_{k=1}^{j-1}c_{k}(x)$ (j=1,2,…,n)*.* Q:[0, 1]→[0, 1] *is a basic unit-interval monotonic (BUM) function, which satisfying: (1)* Q(0)=0*; (2)* Q(1)=1*; (3)* Q(x)≥Q(y) *if* x≥y*.*

As discussed before, existing extensions of PA and POWA are not suitable for aggregation of interval-valued dual hesitant fuzzy information. To help MCGDM methodologies accommodate complicated problems where decision hesitancy and criteria prioritization exist, we here develop two fundamental interval-valued dual hesitant fuzzy prioritized average operators and investigate their desirable properties. 

### 4.1. Interval-Valued Dual Hesitant Fuzzy PA Operator

By extending the conventional PA operator in Definition 6 to accommodate situations where inputs are IVDHFEs, we derive the following interval-valued dual hesitant fuzzy prioritized weighted average (IVDHFPWA) operator.

**Definition** **8.** *For a collection of IVDHFEs* ${\tilde{r}}_{j}(j=1,2,\ldots ,n)$*, which are prioritized so that* ${\tilde{r}}_{j}≺{\tilde{r}}_{j-1}$*, then the IVDHFPWA operator is defined as*

(10) $$IVDHFPWA({\tilde{r}}_{1},{\tilde{r}}_{2},\ldots ,{\tilde{r}}_{n})=\frac{T_{1}}{\sum_{j=1}^{n}T_{j}}{\tilde{r}}_{1}⊕\frac{T_{2}}{\sum_{j=1}^{n}T_{j}}{\tilde{r}}_{2}⊕\ldots ⊕\frac{T_{n}}{\sum_{j=1}^{n}T_{j}}{\tilde{r}}_{n}$$

(11) $$=\overset{n}{\underset{j=1}{⊕}}\left(\frac{T_{j}{\tilde{r}}_{j}}{\sum_{j=1}^{n}T_{j}}\right).$$

*Here* $T_{1}=1$*,* $T_{j}=\prod_{k=1}^{j-1}1-e({\tilde{r}}_{k})=(1-e({\tilde{r}}_{j-1}))T_{j-1}$*, and* $e({\tilde{r}}_{k})$ *is the entropy of* ${\tilde{r}}_{k}$ *as shown in Definition 4.*

Based on the operations of IVDHFEs, IVDHFPWA operator can be rewritten according to the following Theorem 2.

**Theorem** **2.** *Let* ${\tilde{r}}_{j}=\left({\tilde{h}}_{j},{\tilde{g}}_{j}\right)$ *be a collection of IVDHFEs, then the result aggregated from Definition 8 is still an interval-valued dual hesitant fuzzy element, and we have:*

(12) $$IVDHFPWA({\tilde{r}}_{1},{\tilde{r}}_{2},\ldots ,{\tilde{r}}_{n})=\;\cup_{[\mu_{j}^{L},\mu_{j}^{U}]\in {\tilde{h}}_{j},[\nu_{j}^{L},\nu_{j}^{U}]\in {\tilde{g}}_{j}}(\left\{\left[1-\prod_{j=1}^{n}{\left(1-\mu_{j}^{L}\right)}^{\frac{T_{j}}{\sum_{j=1}^{n}T_{j}}},1-\prod_{j=1}^{n}{\left(1-\mu_{j}^{U}\right)}^{\frac{T_{j}}{\sum_{j=1}^{n}T_{j}}}\right]\right\},\;\left\{\left[\prod_{j=1}^{n}{\left(\nu_{j}^{L}\right)}^{\frac{T_{j}}{\sum_{j=1}^{n}T_{j}}},\prod_{j=1}^{n}{\left(\nu_{j}^{U}\right)}^{\frac{T_{j}}{\sum_{j=1}^{n}T_{j}}}\right]\right\}),$$

**Proof.**  Theorem 2 can be proved by mathematical induction method as follows.

(1) When n=1, obviously, Theorem 2 is right.
  $$IVDHFPWA(\tilde{r})=\cup_{[\mu^{L},\mu^{U}]\in \tilde{h},[\nu^{L},\nu^{U}]\in \tilde{g}}\left(\left\{\left[\mu_{j}^{L},\mu_{j}^{U}\right]\right\},\left\{\left[\nu_{j}^{L},\nu_{j}^{U}\right]\right\}\right);$$
(2) When n=2, ${\tilde{r}}_{1}=\cup_{[\mu_{1}^{L},\mu_{1}^{U}]\in {\tilde{h}}_{1},[\nu_{1}^{L},\nu_{1}^{U}]\in {\tilde{g}}_{1}}\left(\left\{\left[\mu_{1}^{L},\mu_{1}^{U}\right]\right\},\left\{\left[\nu_{1}^{L},\nu_{1}^{U}\right]\right\}\right)$,
  $$\frac{T_{1}}{\sum_{j=1}^{n}T_{j}}{\tilde{r}}_{1}=\cup_{[\mu_{1}^{L},\mu_{1}^{U}]\in {\tilde{h}}_{1},[\nu_{1}^{L},\nu_{1}^{U}]\in {\tilde{g}}_{1}}(\left\{\left[1-{\left(1-\mu_{1}^{L}\right)}^{\frac{T_{1}}{\sum_{j=1}^{n}T_{j}}},1-{\left(1-\mu_{1}^{U}\right)}^{\frac{T_{1}}{\sum_{j=1}^{n}T_{j}}}\right]\right\},\;\left\{\left[{\left(\nu_{1}^{L}\right)}^{\frac{T_{1}}{\sum_{j=1}^{n}T_{j}}},{\left(\nu_{1}^{U}\right)}^{\frac{T_{1}}{\sum_{j=1}^{n}T_{j}}}\right]\right\})\;{\tilde{r}}_{2}=\cup_{[\mu_{2}^{L},\mu_{2}^{U}]\in {\tilde{h}}_{2},[\nu_{2}^{L},\nu_{2}^{U}]\in {\tilde{g}}_{2}}\left(\left\{\left[\mu_{2}^{L},\mu_{2}^{U}\right]\right\},\left\{\left[\nu_{2}^{L},\nu_{2}^{U}\right]\right\}\right)\;\frac{T_{2}}{\sum_{j=1}^{n}T_{j}}{\tilde{r}}_{2}=\cup_{[\mu_{2}^{L},\mu_{2}^{U}]\in {\tilde{h}}_{2},[\nu_{2}^{L},\nu_{2}^{U}]\in {\tilde{g}}_{2}}(\left\{\left[1-{\left(1-\mu_{2}^{L}\right)}^{\frac{T_{2}}{\sum_{j=1}^{n}T_{j}}},1-{\left(1-\mu_{2}^{U}\right)}^{\frac{T_{2}}{\sum_{j=1}^{n}T_{j}}}\right]\right\},\;\left\{\left[{\left(\nu_{2}^{L}\right)}^{\frac{T_{2}}{\sum_{j=1}^{n}T_{j}}},{\left(\nu_{2}^{U}\right)}^{\frac{T_{2}}{\sum_{j=1}^{n}T_{j}}}\right]\right\})\;\frac{T_{1}}{\sum_{j=1}^{n}T_{j}}{\tilde{r}}_{1}+\frac{T_{2}}{\sum_{j=1}^{n}T_{j}}{\tilde{r}}_{2}=\cup_{[\mu_{1}^{L},\mu_{1}^{U}]\in {\tilde{h}}_{1},[\mu_{2}^{L},\mu_{2}^{U}]\in {\tilde{h}}_{2},[\nu_{1}^{L},\nu_{1}^{U}]\in {\tilde{g}}_{1},[\nu_{2}^{L},\nu_{2}^{U}]\in {\tilde{g}}_{2}}\;(\left\{\left[1-{\left(1-\mu_{1}^{L}\right)}^{\frac{T_{1}}{\sum_{j=1}^{n}T_{j}}}{\left(1-\mu_{2}^{L}\right)}^{\frac{T_{2}}{\sum_{j=1}^{n}T_{j}}},1-{\left(1-\mu_{1}^{U}\right)}^{\frac{T_{1}}{\sum_{j=1}^{n}T_{j}}}{\left(1-\mu_{2}^{U}\right)}^{\frac{T_{2}}{\sum_{j=1}^{n}T_{j}}}\right]\right\},\;\left\{\left[{\left(\nu_{1}^{L}\right)}^{\frac{T_{1}}{\sum_{j=1}^{n}T_{j}}}{\left(\nu_{2}^{L}\right)}^{\frac{T_{2}}{\sum_{j=1}^{n}T_{j}}},{\left(\nu_{1}^{U}\right)}^{\frac{T_{1}}{\sum_{j=1}^{n}T_{j}}}{\left(\nu_{2}^{U}\right)}^{\frac{T_{2}}{\sum_{j=1}^{n}T_{j}}}\right]\right\}).$$
  So when n=2, Theorem 2 also is right.
(3) Suppose when n=k, Theorem 2 is right, then we have:
  $$IVDHFPWA\left({\tilde{r}}_{1},{\tilde{r}}_{2},\ldots ,{\tilde{r}}_{k}\right)=\cup_{[\mu_{j}^{L},\mu_{j}^{U}]\in {\tilde{h}}_{j},[\nu_{j}^{L},\nu_{j}^{U}]\in {\tilde{g}}_{j}}\;\left\{\left\{\left[1-\prod_{j=1}^{k}{\left(1-\mu_{j}^{L}\right)}^{\frac{T_{1}}{\sum_{j=1}^{k}T_{j}}},1-\prod_{j=1}^{k}{\left(1-\mu_{j}^{U}\right)}^{\frac{T_{1}}{\sum_{j=1}^{k}T_{j}}}\right]\right\},\left\{\left[\prod_{j=1}^{k}{\left(\nu_{j}^{L}\right)}^{\frac{T_{1}}{\sum_{j=1}^{k}T_{j}}},\prod_{j=1}^{k}{\left(\nu_{j}^{U}\right)}^{\frac{T_{1}}{\sum_{j=1}^{k}T_{j}}}\right]\right\}\right\},\;w_{k+1}{\tilde{r}}_{k+1}=\;\cup_{[\mu_{1}^{L},\mu_{1}^{U}]\in {\tilde{h}}_{1},[\nu_{1}^{L},\nu_{1}^{U}]\in {\tilde{g}}_{1}}\left(\left\{\left[1-{\left(1-\mu_{1}^{L}\right)}^{\frac{T_{k+1}}{\sum_{j=1}^{k+1}T_{j}}},1-{\left(1-\mu_{1}^{U}\right)}^{\frac{T_{k+1}}{\sum_{j=1}^{k+1}T_{j}}}\right]\right\},\left\{\left[{\left(\nu_{1}^{L}\right)}^{\frac{T_{k+1}}{\sum_{j=1}^{k+1}T_{j}}},{\left(\nu_{1}^{U}\right)}^{\frac{T_{k+1}}{\sum_{j=1}^{k+1}T_{j}}}\right]\right\}\right).$$

Then when n=k+1, we have

$$IVDHFPWA\left({\tilde{r}}_{1},{\tilde{r}}_{2},\ldots ,{\tilde{r}}_{k+1}\right)=\left(\overset{k}{\underset{j=1}{⊕}}w_{j}{\tilde{r}}_{j}\right)⊕w_{k+1}{\tilde{r}}_{k+1}=\cup_{[\mu_{j}^{L},\mu_{j}^{U}]\in {\tilde{h}}_{j},[\nu_{j}^{L},\nu_{j}^{U}]\in {\tilde{g}}_{j}}\;\left\{\left\{\left[1-\prod_{j=1}^{k+1}{\left(1-\mu_{j}^{L}\right)}^{\frac{T_{j}}{\sum_{j=1}^{k+1}T_{j}}},1-\prod_{j=1}^{k+1}{\left(1-\mu_{j}^{U}\right)}^{\frac{T_{j}}{\sum_{j=1}^{k+1}T_{j}}}\right]\right\},\left\{\left[\prod_{j=1}^{k+1}{\left(\nu_{j}^{L}\right)}^{\frac{T_{j}}{\sum_{j=1}^{k+1}T_{j}}},\prod_{j=1}^{k+1}{\left(\nu_{j}^{U}\right)}^{\frac{T_{j}}{\sum_{j=1}^{k+1}T_{j}}}\right]\right\}\right\}.$$

So when n=k+1, Theorem 2 is right too.

According to steps (1)–(3), we can conclude that Theorem 2 is right for all n.

Furthermore, it can be easily proved that the IVDHFPWA operator has the following properties.

**Theorem** **3.** *(Idempotency) Let* ${\tilde{r}}_{j}=\left({\tilde{h}}_{j},{\tilde{g}}_{j}\right)(j=1,2,\ldots ,n)$ *be a collection of IVDHFEs. If* ${\tilde{r}}_{j}=\tilde{r}=(\tilde{h},\tilde{g})$ *for all* j=1,2,…,n*, then:*

$$IVDHFPWA({\tilde{r}}_{1},{\tilde{r}}_{2},\ldots ,{\tilde{r}}_{n})=\tilde{r}=(\tilde{h},\tilde{g}).$$

**Theorem** **4.** *(Boundedness) Let* ${\tilde{r}}_{j}=\left({\tilde{h}}_{j},{\tilde{g}}_{j}\right)(j=1,2,\ldots ,n)$ *be a collection of IVDHFEs, then IVDHFPWA operator lie between the max and min values,*

$${\tilde{r}}^{-}\leq IVDHFPWA({\tilde{r}}_{1},{\tilde{r}}_{2},\ldots ,{\tilde{r}}_{n})\leq {\tilde{r}}^{+},$$

*where*

$${\tilde{r}}^{-}=\left(\left[\underset{j}{\min}\;\mu_{j}^{L},\underset{j}{\min}\;\mu_{j}^{U}\right],\left[\underset{j}{\max}\;\nu_{j}^{L},\underset{j}{\max}\;\nu_{j}^{U}\right]\right)\;\mathrm{and}\;{\tilde{r}}^{+}=\left(\left[\underset{j}{\max}\;\mu_{j}^{L},\underset{j}{\max}\;\mu_{j}^{U}\right],\left[\underset{j}{\min}\;\nu_{j}^{L},\underset{j}{\min}\;\nu_{j}^{U}\right]\right).$$

**Theorem** **5.** *(Commutativity) Let* ${\tilde{r}}_{j}=\left({\tilde{h}}_{j},{\tilde{g}}_{j}\right)(j=1,2,\ldots ,n)$ *be a collection of IVDHFEs,* $\left({\tilde{r}}_{1}{}^{*},{\tilde{r}}_{2}{}^{*},\ldots ,{\tilde{r}}_{n}{}^{*}\right)$ *be any a permutation of* $\left({\tilde{r}}_{1},{\tilde{r}}_{2},\ldots ,{\tilde{r}}_{n}\right)$*, then,*

$$IVDHFPWA({\tilde{r}}_{1}{}^{*},{\tilde{r}}_{2}{}^{*},\ldots ,{\tilde{r}}_{n}{}^{*})=IVDHFPWA({\tilde{r}}_{1},{\tilde{r}}_{2},\ldots ,{\tilde{r}}_{n}).$$

**Example** **2.** *Suppose a collection of IVDHFEs:* ${\tilde{r}}_{1}=\left(\left\{[0.1,0.3],[0.3,0.4]\},\{[0.3,0.4],[0.4,0.5]\right\}\right)$*,* ${\tilde{r}}_{2}=\left(\left\{[0.3,0.4],[0.5,0.7]\},\{[0.1,0.2]\right\}\right)$*,* ${\tilde{r}}_{3}=\left(\left\{[0.4,0.5]\},\{[0.3,0.4],[0.4,0.5]\right\}\right)$*,* ${\tilde{r}}_{4}=\left(\left\{[0.1,0.3]\},\{[0.6,0.7]\right\}\right)$*. Then according to Definition 8, we can obtain* w=(0.6655,0.2163,0.1027,0.0154)*, and the overall aggregation result* $\tilde{r}$ *is*

$$\tilde{r}=IVDHFPWA\left({\tilde{r}}_{1},{\tilde{r}}_{2},{\tilde{r}}_{3},{\tilde{r}}_{4}\right)\;=(\{[0.1824,0.346],[0.2398,0.437],[0.3083,0.4097],[0.3569,0.4919]\},\;\left\{[0.2391,0.3473],[0.2463,0.3553],[0.2895,0.4029],[0.2982,0.4122]\right\}).$$

*Further, according to Equation (2), we obtain the score of* $\tilde{r}$ *is* $s(\tilde{r})=0.0226$*.*

### 4.2. Interval-Valued Dual Hesitant Fuzzy POWA Operator

In this subsection, by extending the POWA operator shown in Definition 7 to interval-valued dual hesitant fuzzy environments, we propose the interval-valued dual hesitant fuzzy prioritized ordered weighted average (IVDHFPOWA) operator.

**Definition** **9.** *For a collection of IVDHFEs* ${\tilde{r}}_{j}(j=1,2,\ldots ,n)$*, with the prioritized relationship* ${\tilde{r}}_{j}≺{\tilde{r}}_{j-1}$*, then the IVDHFPOWA operator is defined as follows:*

(13) $$IVDHFPOWA({\tilde{r}}_{1},{\tilde{r}}_{2},\ldots ,{\tilde{r}}_{n})=\sum_{j=1}^{n}w_{j}{\tilde{r}}_{ind(j)}$$

(14) $$=\sum_{j=1}^{n}\left(f\left(\frac{\sum_{k=1}^{j}T_{ind(k)}}{\sum_{j=1}^{n}T_{j}}\right)-f\left(\frac{\sum_{k=1}^{j-1}T_{ind(k)}}{\sum_{j=1}^{n}T_{j}}\right)\right){\tilde{r}}_{ind(k)}.$$

*Here* ind(j) *be the index of the* j*th most satisfied criterion that holds the* j*th largest of* ${\tilde{r}}_{j}(j=1,2,\ldots ,n)$*.* $T_{1}=1$*,* $T_{\sigma (0)}=0$*,* $T_{j}=\prod_{k=1}^{j}1-e({\tilde{r}}_{k})=(1-e({\tilde{r}}_{j-1}))T_{j-1}$*,* $e({\tilde{r}}_{k})$ *is the entropy measure of* ${\tilde{r}}_{k}$ *as shown in Definition 4.* Q:[0, 1]→[0, 1] *is a basic unit-interval monotonic (BUM) function, which satisfies the properties: (1)* Q(0)=0*; (2)* Q(1)=1*; (3)* Q(x)≥Q(y) *if* x≥y*.*

Similar to Theorem 1, we obtain the following Theorem 5.

**Theorem** **6.** *Let* ${\tilde{r}}_{j}=\left({\tilde{h}}_{j},{\tilde{g}}_{j}\right)$ *be a collection of IVDHFEs,* ${\tilde{r}}_{ind(j)}=\left({\tilde{h}}_{ind(j)},{\tilde{g}}_{ind(j)}\right)$ *be the reordered collection of* ${\tilde{r}}_{j}$*, then the result obtained from Definition 9 is still an interval-valued dual hesitant fuzzy element, and we have*

(15) $$IVDHFPOWA({\tilde{r}}_{1},{\tilde{r}}_{2},\ldots ,{\tilde{r}}_{n})=\;\cup_{[\mu_{ind(j)}^{L},\mu_{ind(j)}^{U}]\in {\tilde{h}}_{ind(j)},[\nu_{ind(j)}^{L},\nu_{ind(j)}^{U}]\in {\tilde{g}}_{ind(j)}}(\left\{\left[1-\prod_{j=1}^{n}{\left(1-\mu_{ind(j)}^{L}\right)}^{w_{j}},1-\prod_{j=1}^{n}{\left(1-\mu_{ind(j)}^{U}\right)}^{w_{j}}\right]\right\},\;\left\{\left[\prod_{j=1}^{n}{\left(\nu_{ind(j)}^{L}\right)}^{w_{j}},\prod_{j=1}^{n}{\left(\nu_{ind(j)}^{U}\right)}^{w_{j}}\right]\right\}),$$

*where*

(16) $$w_{j}=f\left(\frac{\sum_{k=1}^{j}T_{ind(k)}}{\sum_{j=1}^{n}T_{j}}\right)-f\left(\frac{\sum_{k=1}^{j-1}T_{ind(k)}}{\sum_{j=1}^{n}T_{j}}\right).$$

Theorem 6 can be proved by mathematical induction method similar to Theorem 2, and the steps in details are omitted here. Also similar to Theorems 3–5, it can be easily proved that IVDHFPOWA operator has the following properties.

**Theorem** **7.** *(Idempotency) Let* ${\tilde{r}}_{j}=\left({\tilde{h}}_{j},{\tilde{g}}_{j}\right)$ (j=1,2,…,n) *be a collection of IVDHFEs. If* ${\tilde{r}}_{j}=\tilde{r}=(\tilde{h},\tilde{g})$ *for all* j=1,2,…,n*,* j=1,2,…,n *then: missing equation numbers*

$$IVDHFPOWA({\tilde{r}}_{1},{\tilde{r}}_{2},\ldots ,{\tilde{r}}_{n})=\tilde{r}=(\tilde{h},\tilde{g}).$$

**Theorem** **8.** *(Boundedness) Let* ${\tilde{r}}_{j}=\left({\tilde{h}}_{j},{\tilde{g}}_{j}\right)(j=1,2,\ldots ,n)$ *be a collection of IVDHFEs, then IVDHFPWA operator lie between the max value* ${\tilde{r}}^{+}$ *and min value* ${\tilde{r}}^{-}$ *, that is,*

$${\tilde{r}}^{-}\leq IVDHFPOWA({\tilde{r}}_{1},{\tilde{r}}_{2},\ldots ,{\tilde{r}}_{n})\leq {\tilde{r}}^{+},$$

*where* ${\tilde{r}}^{-}=\left(\left[\underset{j}{\min}\;\mu_{j}^{L},\underset{j}{\min}\;\mu_{j}^{U}\right],\left[\underset{j}{\max}\;\nu_{j}^{L},\underset{j}{\max}\;\nu_{j}^{U}\right]\right)$ *and* ${\tilde{r}}^{+}=\left(\left[\underset{j}{\max}\;\mu_{j}^{L},\underset{j}{\max}\;\mu_{j}^{U}\right],\left[\underset{j}{\min}\;\nu_{j}^{L},\underset{j}{\min}\;\nu_{j}^{U}\right]\right)$*.*

**Theorem** **9.** *(Commutativity) Let* ${\tilde{r}}_{j}=\left({\tilde{h}}_{j},{\tilde{g}}_{j}\right)(j=1,2,\ldots ,n)$ *be a collection of IVDHFEs,* $\left({\tilde{r}}_{1}{}^{*},{\tilde{r}}_{2}{}^{*},\ldots ,{\tilde{r}}_{n}{}^{*}\right)$ *be any a permutation of* $\left({\tilde{r}}_{1},{\tilde{r}}_{2},\ldots ,{\tilde{r}}_{n}\right)$*, then we have*

$$IVDHFPOWA({\tilde{r}}_{1}{}^{*},{\tilde{r}}_{2}{}^{*},\ldots ,{\tilde{r}}_{n}{}^{*})=IVDHFPOWA({\tilde{r}}_{1},{\tilde{r}}_{2},\ldots ,{\tilde{r}}_{n}).$$

Additionally, as for the relationship between operators IVDHFPWA and IVDHFPOWA, we have the following Theorem 10.

**Theorem** **10.** *If* ${\tilde{r}}_{ind(j)}={\tilde{r}}_{j}$ *for all* j=1,2,…,n*, then the IVDHFPOWA operator reduces to the IVDHFPWA operator.*

**Example** **3.** *Suppose a collection of IVDHFEs,* ${\tilde{r}}_{1}=\left(\left\{[0.1,0.3],[0.3,0.4]\},\{[0.3,0.4],[0.4,0.5]\right\}\right)$*,* ${\tilde{r}}_{2}=\left(\left\{[0.3,0.4],[0.5,0.7]\},\{[0.1,0.2]\right\}\right)$*,* ${\tilde{r}}_{3}=\left(\left\{[0.4,0.5]\},\{[0.3,0.4],[0.4,0.5]\right\}\right)$*,* ${\tilde{r}}_{4}=\left(\left\{[0.1,0.3]\},\{[0.6,0.7]\right\}\right)$*. According to Definition 9, we firstly have the reordered collection of IVDHFEs as:* ${\tilde{r}}_{ind(1)}=\left(\left\{[0.4,0.5]\},\{[0.3,0.4],[0.4,0.5]\right\}\right)$*,* ${\tilde{r}}_{ind(2)}=\left(\left\{[0.3,0.4],[0.5,0.7]\},\{[0.1,0.2]\right\}\right)$*,* ${\tilde{r}}_{ind(3)}=\left(\left\{[0.1,0.3]\},\{[0.6,0.7]\right\}\right)$*, and* ${\tilde{r}}_{ind(4)}=\left(\left\{[0.1,0.3],[0.3,0.4]\},\{[0.3,0.4],[0.4,0.5]\right\}\right)$*. Then, let the basic unit-interval monotonic (BUM) function be* $f=x^{2}$*, we obtain* w=(0.6366,0.2053,0.1076,0.0505)*. Subsequently, the overall aggregation result* $\tilde{r}$ *is calculated as*

$$\tilde{r}=IVDHFPOWA\left({\tilde{r}}_{1},{\tilde{r}}_{2},{\tilde{r}}_{3},{\tilde{r}}_{4}\right)=(\{[0.3397,0.4526],[0.348,0.4568],[0.3838,0.5252],[0.3916,0.5289]\},\;\left\{[0.258,0.3685],[0.2617,0.3726],[0.3098,0.4247],[0.3143,0.4295]\right\}).$$

*Further, according to Equation (2), we have the score of* $\tilde{r}$ *as* $s(\tilde{r})=0.0859$*.*

## 5. Approaches for ERSE with Decision Hesitancy and Prioritization among Criteria

On the strength of IVDHFS and the above-developed operators, i.e., IVDHFPWA and IVDHFPOWA, now we construct effective MCGDM approaches to tackle complicate ERSE problems in which prioritization relationships exists among criteria and decision hesitancy occurs when decision makers give their uncertain assessments under those criteria. 

Let $X=\{x_{1},\ldots ,x_{i},\ldots ,x_{n}\}$ be the set of response solutions, $C=\{C_{1},\ldots ,C_{j},\ldots ,C_{m}\}$ be the set of criteria, and $E=\{E_{1},\ldots ,E_{k},\ldots ,E_{t}\}$ be the set of decision makers. Suppose that, according to knowledge from decision contexts, decision makers reach a prioritization relationship among these criteria: $C_{\sigma (1)}≻\ldots ≻C_{\sigma (j)}≻\ldots ≻C_{\sigma (m)}$, which means criterion $C_{\sigma (j-1)}$ have a higher priority level than criterion $C_{\sigma (j)}$. Suppose $R^{\left(k\right)}={\left({\tilde{r}}_{ij}^{\left(k\right)}\right)}_{n\times m}$ (k=1,2,…,t) be the individual interval-valued dual hesitant fuzzy (IVDHF) decision matrices, and ${\tilde{r}}_{ij}^{\left(k\right)}$ denotes the assessment given by kth decision maker with respect to the alternative $x_{i}$ under the criterion $C_{j}$. 

Subsequently, in view of the fact weighting information for decision makers usually cannot always be determined appropriately beforehand in complicate group decision making situations, we develop the following Approach I for situations with known weights of decision makers, i.e., $\lambda =\{\lambda_{1},\ldots ,\lambda_{k},\ldots ,\lambda_{t}\}$, and the following Approach II for other situations where decision makers’ weights cannot be obtained in advance.

**Approach** **I.** **Interval-valued dual hesitant fuzzy MCGDM with prioritization among criteria and known decision makers’ weights**

**Step I-1.** Transform each individual IVDHF decision matrices ${\tilde{R}}^{\left(k\right)}={\left({\tilde{r}}_{ij}^{\left(k\right)}\right)}_{n\times m}={\left({\tilde{h}}_{ij}^{\left(k\right)},{\tilde{g}}_{ij}^{\left(k\right)}\right)}_{n\times m}$ (k=1,2,…,t) to the prioritized individual IVDHF decision matrices ${\bar{\tilde{R}}}^{\left(k\right)}={\left({\bar{\tilde{r}}}_{ij}^{\left(k\right)}\right)}_{n\times m}={\left({\bar{\tilde{h}}}_{ij}^{\left(k\right)},{\bar{\tilde{g}}}_{ij}^{\left(k\right)}\right)}_{n\times m}$ (k=1,2,…,t) according to priority levels of criteria, where ${\bar{\tilde{h}}}_{ij}^{\left(k\right)}=\cup_{{\bar{\tilde{\mu }}}_{ij}^{k}\in {\bar{\tilde{h}}}_{ij}^{\left(k\right)}}\left\{{\bar{\tilde{\mu }}}_{ij}^{k}\right\}$, ${\bar{\tilde{g}}}_{ij}^{\left(k\right)}=\cup_{{\bar{\tilde{\nu }}}_{ij}^{k}\in {\bar{\tilde{g}}}_{ij}^{\left(k\right)}}\left\{{\bar{\tilde{\nu }}}_{ij}^{k}\right\}$.

**Step I-2.** Calculate prioritized levels in prioritized individual IVDHF decision matrices. 

Derive the entropy values of ${\bar{\tilde{r}}}_{ij}^{\left(k\right)}$ according to Equations (4) and (5), then compute the numerical prioritized levels $T_{ij}^{\left(k\right)}(i=1,2,\ldots ,n;j=1,2,\ldots ,m;k=1,2,\ldots ,t)$ in each prioritized individual IVDHF decision matrix, where

(17) $$T_{ij}^{\left(k\right)}=\prod_{l=1}^{j-1}1-e({\bar{\tilde{r}}}_{il}^{\left(k\right)})=(1-e({\bar{\tilde{r}}}_{i(j-1)}^{\left(k\right)}))T_{i,(j-1)}^{\left(k\right)},$$

(18) $$T_{i1}^{\left(k\right)}=1.$$

**Step I-3.** Obtain aggregated results in prioritized individual IVDHF decision matrix by applying operator IVDHFPWA, or operator IVDHFPOWA. Utilize the IVDHFPWA operator described in Definition 8 to aggregate ${\bar{\tilde{r}}}_{ij}^{\left(k\right)}$ so that we get the *k*th decision maker’s decision result ${\tilde{r}}_{i}^{\left(k\right)}$ on the alternative $x_{i}$, where

(19) $${\tilde{r}}_{i}^{\left(k\right)}=IVDHFPWA({\bar{\tilde{r}}}_{i1}^{\left(k\right)},\ldots ,{\bar{\tilde{r}}}_{ij}^{\left(k\right)},\ldots ,{\bar{\tilde{r}}}_{im}^{\left(k\right)})=\;\cup_{[{\bar{\mu }}_{ij}^{Lk},{\bar{\mu }}_{ij}^{Uk}]\in {\bar{\tilde{h}}}_{ij}^{\left(k\right)},[{\bar{\nu }}_{ij}^{Lk},{\bar{\nu }}_{ij}^{Uk}]\in {\bar{\tilde{g}}}_{ij}^{\left(k\right)}}(\left\{\left[1-\prod_{j=1}^{m}{\left(1-{\bar{\mu }}_{ij}^{Lk}\right)}^{\frac{T_{ij}^{\left(k\right)}}{\sum_{j=1}^{m}T_{ij}^{\left(k\right)}}},1-\prod_{j=1}^{m}{\left(1-{\bar{\mu }}_{ij}^{Uk}\right)}^{\frac{T_{ij}^{\left(k\right)}}{\sum_{j=1}^{m}T_{ij}^{\left(k\right)}}}\right]\right\},\;\left\{\left[\prod_{j=1}^{m}{\left(\nu_{ij}^{Lk}\right)}^{\frac{T_{ij}^{\left(k\right)}}{\sum_{j=1}^{m}T_{ij}^{\left(k\right)}}},\prod_{j=1}^{m}{\left(\nu_{ij}^{Uk}\right)}^{\frac{T_{ij}^{\left(k\right)}}{\sum_{j=1}^{m}T_{ij}^{\left(k\right)}}}\right]\right\}).$$

Or utilize the IVDHFPOWA operator described in Definition 9, then we have:

(20) $${\tilde{r}}_{i}^{\left(k\right)}=IVDHFPOWA({\bar{\tilde{r}}}_{i1}^{\left(k\right)},\ldots ,{\bar{\tilde{r}}}_{ij}^{\left(k\right)},\ldots ,{\bar{\tilde{r}}}_{im}^{\left(k\right)})=\sum_{j=1}^{m}w_{ij}^{k}{\tilde{r}}_{iind(j)}^{\left(k\right)}\;\cup_{[\mu_{iind(j)}^{Lk},\mu_{iind(j)}^{Uk}]\in {\tilde{h}}_{iind(j)}^{\left(k\right)},[\nu_{iind(j)}^{Lk},\nu_{iind(j)}^{Uk}]\in {\tilde{g}}_{iind(j)}^{\left(k\right)}}(\left\{\left[1-\prod_{j=1}^{m}{\left(1-\mu_{iind(j)}^{Lk}\right)}^{w_{ij}^{k}},1-\prod_{j=1}^{m}{\left(1-\mu_{iind(s)}^{Uk}\right)}^{w_{ij}^{k}}\right]\right\},\;\left\{\left[\prod_{j=1}^{m}{\left(\nu_{iind(j)}^{Lk}\right)}^{w_{ij}^{k}},\prod_{j=1}^{m}{\left(\nu_{iind(s)}^{Uk}\right)}^{w_{ij}^{k}}\right]\right\}),$$

where

(21) $$w_{ij}^{k}=f\left(\frac{\sum_{l=1}^{j}T_{iind(l)}^{k}}{\sum_{j=1}^{m}T_{ij}^{k}}\right)-f\left(\frac{\sum_{l=1}^{j-1}T_{iind(l)}^{k}}{\sum_{j=1}^{m}T_{ij}^{k}}\right).$$

**Step I-4.** Obtain collective results of all alternatives by applying weights of decision makers. 

Suppose the weighting vector for decision makers be $\lambda =\{\lambda_{1},\ldots ,\lambda_{k},\ldots ,\lambda_{t}\}$, then we aggregate all individual overall decision values ${\tilde{r}}_{i}^{\left(k\right)}(k=1,2,\ldots ,t)$ into the overall group decision values ${\tilde{r}}_{i}(i=1,2,\ldots ,n)$ by use of IVDHFWA operator described in Definition 5, where

(22) $${\tilde{r}}_{i}=\overset{t}{\underset{k=1}{⊕}}(\lambda_{k}{\tilde{r}}_{i}^{\left(k\right)})=\cup_{[\mu_{i}^{Lk},\mu_{i}^{Uk}]\in {\tilde{h}}_{i}^{\left(k\right)},[\nu_{i}^{Lk},\nu_{i}^{Uk}]\in {\tilde{g}}_{i}^{\left(k\right)}}\{\left\{\left[1-\prod_{k=1}^{t}{\left(1-\mu_{i}^{Lk}\right)}^{\lambda_{k}},1-\prod_{k=1}^{t}{\left(1-\mu_{i}^{Uk}\right)}^{\lambda_{k}}\right]\right\},\;\left\{\left[\prod_{k=1}^{t}{\left(\nu_{i}^{Lk}\right)}^{\lambda_{k}},\prod_{k=1}^{t}{\left(\nu_{i}^{Uk}\right)}^{\lambda_{k}}\right]\right\}\}.$$

**Step I-5.** Determine the ranking order of all alternatives. 

For each alternative, according to Equations (2) and (3) respectively, we calculate the score value $s({\tilde{r}}_{i})$ and accuracy value $v({\tilde{r}}_{i})$ based on its group decision value ${\tilde{r}}_{i}$. Then according to the comparative method introduced by Ju, Liu and Yang [31], we can determine the ranking order of all alternatives. 

More practically, due to ill-structured problem definitions in complex scenarios, weighting vector for decision makers usually cannot be appropriately determined in advance, so we develop the following Approach II to address these complicate situations.

**Approach** **II.** **Interval-valued dual hesitant fuzzy MCGDM with prioritization among criteria but unknown decision makers’ weights**

**Step II-1.** See Step I-1.

**Step II-2.** Obtain prioritized interval-valued dual hesitant fuzzy group decision matrix. 

By combining each prioritized individual interval-valued dual hesitant fuzzy decision matrix ${\bar{\tilde{R}}}^{\left(k\right)}={\left({\bar{\tilde{r}}}_{ij}^{\left(k\right)}\right)}_{n\times m}={\left({\bar{\tilde{h}}}_{ij}^{\left(k\right)},{\bar{\tilde{g}}}_{ij}^{\left(k\right)}\right)}_{n\times m}(k=1,2,\ldots ,t)$ into the prioritized interval-valued dual hesitant fuzzy group decision matrix:

$$\bar{\tilde{R}}={\left({\bar{\tilde{r}}}_{ij}\right)}_{n\times m}={\left({\bar{\tilde{h}}}_{ij},{\bar{\tilde{g}}}_{ij}\right)}_{n\times m},$$

where ${\bar{\tilde{h}}}_{ij}=\cup_{{\bar{\tilde{\mu }}}_{ij}\in {\bar{\tilde{h}}}_{ij}}\left\{{\bar{\tilde{\mu }}}_{ij}\right\}$ and ${\bar{\tilde{g}}}_{ij}=\cup_{{\bar{\tilde{\nu }}}_{ij}\in {\bar{\tilde{g}}}_{ij}}\left\{{\bar{\tilde{\nu }}}_{ij}\right\}$. 

**Step II-3.** Calculate numerical prioritized levels in group decision matrix. 

Derive the entropy values of ${\bar{\tilde{r}}}_{ij}$ according to Equations (4) and (5), then derive the prioritized levels $T_{ij}(i=1,2,\ldots ,n;j=1,2,\ldots ,m)$ in the prioritized group interval-valued dual hesitant fuzzy decision matrix $\bar{\tilde{R}}$, where

(23) $$T_{ij}=\prod_{l=1}^{j-1}1-e({\bar{\tilde{r}}}_{il})=(1-e({\bar{\tilde{r}}}_{i,(j-1)}))T_{i,(j-1)},$$

(24) $$T_{i1}=1.$$

**Step II-4.** Acquire collective decision values of all alternatives by applying the aggregation operator IVDHFPWA or the operator IVDHFPOWA. 

Utilize the IVDHFPWA operator proposed in Definition 8 to aggregate ${\bar{\tilde{r}}}_{ij}$ so that we get the group decision result ${\tilde{r}}_{i}$ on the alternative $x_{i}$, where

(25) $${\tilde{r}}_{i}=IVDHFPWA({\bar{\tilde{r}}}_{i1},\ldots ,{\bar{\tilde{r}}}_{ij},\ldots ,{\bar{\tilde{r}}}_{im})=\;\cup_{[{\bar{\mu }}_{ij}^{L},{\bar{\mu }}_{ij}^{U}]\in {\bar{\tilde{h}}}_{ij}^{},[{\bar{\nu }}_{ij}^{L},{\bar{\nu }}_{ij}^{U}]\in {\bar{\tilde{g}}}_{ij}^{}}(\left\{\left[1-\prod_{j=1}^{m}{\left(1-{\bar{\mu }}_{ij}^{L}\right)}^{\frac{T_{ij}^{}}{\sum_{j=1}^{m}T_{ij}^{}}},1-\prod_{j=1}^{m}{\left(1-{\bar{\mu }}_{ij}^{U}\right)}^{\frac{T_{ij}^{}}{\sum_{j=1}^{m}T_{ij}^{}}}\right]\right\},\;\left\{\left[\prod_{j=1}^{m}{\left(\nu_{ij}^{L}\right)}^{\frac{T_{ij}^{}}{\sum_{j=1}^{m}T_{ij}^{}}},\prod_{j=1}^{m}{\left(\nu_{ij}^{U}\right)}^{\frac{T_{ij}^{}}{\sum_{j=1}^{m}T_{ij}^{}}}\right]\right\}).$$

Or by applying the IVDHFPOWA operator proposed in Definition 9, we have

(26) $${\tilde{r}}_{i}^{}=IVDHFPOWA({\bar{\tilde{r}}}_{i1}^{},\ldots ,{\bar{\tilde{r}}}_{is}^{},\ldots ,{\bar{\tilde{r}}}_{ij}^{})=\;\sum_{j=1}^{m}w_{j}^{}{\tilde{r}}_{i,ind(j)}^{}\cup_{[\mu_{i,ind(j)}^{L},\mu_{i,ind(j)}^{U}]\in {\tilde{h}}_{i,ind(j)}^{},[\nu_{i,ind(j)}^{L},\nu_{i,ind(j)}^{U}]\in {\tilde{g}}_{i,ind(j)}^{}}(\left\{\left[1-\prod_{j=1}^{m}{\left(1-\mu_{i,ind(j)}^{L}\right)}^{w_{j}^{}},1-\prod_{j=1}^{m}{\left(1-\mu_{i,ind(j)}^{U}\right)}^{w_{j}^{}}\right]\right\},\;\left\{\left[\prod_{j=1}^{m}{\left(\nu_{i,ind(j)}^{L}\right)}^{w_{j}^{}},\prod_{j=1}^{m}{\left(\nu_{i,ind(j)}^{U}\right)}^{w_{j}^{}}\right]\right\}),$$

where

(27) $$w_{j}^{}=f\left(\frac{\sum_{l=1}^{j}T_{i,ind(l)}^{}}{\sum_{j=1}^{m}T_{ij}^{}}\right)-f\left(\frac{\sum_{l=1}^{j-1}T_{i,ind(l)}^{}}{\sum_{j=1}^{m}T_{ij}^{}}\right).$$

**Step II-5.** Determine the ranking order of all alternatives. 

For each alternative, according to Equations (2) and (3) respectively, we firstly calculate the score value $s({\tilde{r}}_{i})$ and accuracy value $v({\tilde{r}}_{i})$ based on its ${\tilde{r}}_{i}$. Then according to the comparative method introduced by Ju, Liu and Yang [31], we can determine the ranking order of all alternatives. 

**Figure 1:**
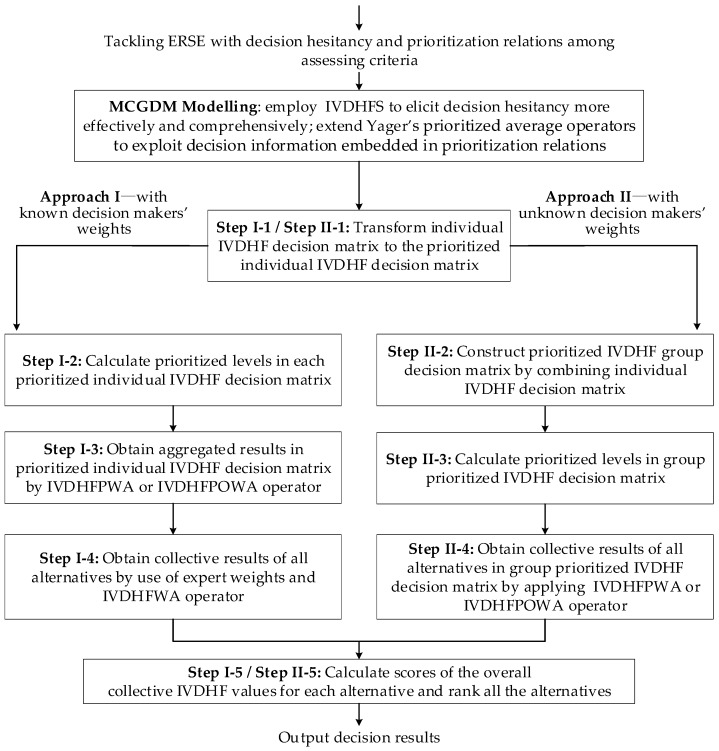
Flowcharts of the proposed Approach I and Approach II.

For more clarity, following Figure 1 delineates the flowcharts of above-developed Approach I and Approach II. 

## 6. Illustrative Example and Comparative Study

### 6.1. Illustrative Example

As pointed out formerly, environmental problems and resulting public health threats that accompany economic development projects have been urging governments to include ERSE in their governance of sustainable development [1,9,13,14,15]. Precedent AHP-based or ANP-based methods [1,14,15] for ERSE generally needs multi-rounds consistency adjustments to derive criteria weights while neglecting the fact that decision makers are usually able to quickly reach a consensus on an rather accurate prioritization relation among assessment criteria. Regarding ERSE with decision hesitancy resulting from problem complexity, the existing ideal solutions-based decision-making methods [9] adopted the distance measure that grounds on subjectively supplementing mechanism for IVDHFEs, thus restricted themselves to situations where decision makers are capable of or willing to specify their pessimistic or optimistic attitudes. Therefore, aiming at the currently existing gap, we have developed the above Approach I and Approach II. 

Now, in this section, we conduct case study to demonstrate practicality and effectiveness of our proposed approaches. Subsequently, we take the assessing criteria listed in Table 1 and the first emergency event shown in Table 2. That is, suppose three emergency response solutions be under evaluation, i.e., $x_{i}(i=1,2,3)$, according to eight criteria $C_{j}(j=1,2,\ldots ,8)$: (1) $C_{1}$—Response time to start the emergency response solution; (2) $C_{2}$—Reasonable organizational structure and clear awareness of responsibilities; (3) $C_{3}$—Economic cost; (4) $C_{4}$—Operability of the response solution; (5) $C_{5}$—Monitoring and forecasting potential hazards; (6) $C_{6}$—Reconstruction ability; (7) $C_{7}$—Social impact; (8) $C_{8}$—Environmental impact. A panel of decision makers, $E_{k}(k=1,2,3)$, have been already organized, and the decision makers have made a consensus opinion on the prioritization relations among the above eight criteria, that is, ($C_{2}$) ≻ ($C_{5}$) ≻ ($C_{1}$) ≻ ($C_{8}$) ≻ ($C_{4}$) ≻ ($C_{6}$) ≻ ($C_{7}$) ≻ ($C_{3}$). Next, all three decision makers $E_{k}(k=1,\;2,\;3)$ were invited to provide their preferences in the form of interval-valued dual hesitant fuzzy elements. Then, three interval-valued dual hesitant fuzzy (IVDHF) decision matrices, i.e., ${\tilde{R}}^{\left(k\right)}={\left({\tilde{r}}_{ij}^{\left(k\right)}\right)}_{3\times 8}$ (k=1, 2, 3), have been collected in Table 4, Table 5 and Table 6. 

Now, by use *of* Matlab^®^-2009a software (The MathWorks, Inc., Natick, MA, USA), we apply our proposed Approach I and Approach II to obtain the ranking order of the three emergency response solutions under different decision making situations with or without weights of decision makers, respectively. For Approach I, suppose the known weighting vector for all three decision makers is λ=[0.3,0.2,0.5]. Decision making steps are detailed as follows.

#### 6.1.1. Decision Making Steps of Approach I

**Step I-1.** Transform each individual IVDHF decision matrix ${\tilde{R}}^{\left(k\right)}={\left({\tilde{r}}_{ij}^{\left(k\right)}\right)}_{3\times 8}={\left({\tilde{h}}_{ij}^{\left(k\right)},{\tilde{g}}_{ij}^{\left(k\right)}\right)}_{3\times 8}$ into the prioritized individual IVDHF decision matrix ${\bar{\tilde{R}}}^{\left(k\right)}={\left({\bar{\tilde{r}}}_{ij}^{\left(k\right)}\right)}_{3\times 8}={\left({\bar{\tilde{h}}}_{ij}^{\left(k\right)},{\bar{\tilde{g}}}_{ij}^{\left(k\right)}\right)}_{3\times 8}(k=1,2,3)$ according to the different priority levels of criteria, as listed in Table 7, Table 8 and Table 9. 

**Step I-2.** Calculate the $e({\bar{\tilde{r}}}_{ij}^{\left(k\right)})$ by Equations (4) and (5), and then obtain the numerical prioritized levels $T_{ij}^{\left(k\right)}$ (i=1,2,3;j=1,2,…,8;k=1,2,3) by Equations (17) and (18) in each prioritized individual decision matrix, where

$$T^{\left(1\right)}=\left[T_{ij}^{\left(1\right)}\right]=\left[\begin{array}{cccccccc} 1 & 0.5 & 0.2 & 0.02 & 0.007 & 0.0038 & 0.0023 & 0.0002\\ 1 & 0.4 & 0.1 & 0.035 & 0.0105 & 0.0021 & 0.000315 & 0.00014\\ 1 & 0.55 & 0.22 & 0.11 & 0.0275 & 0.0165 & 0.0099 & 0.002 \end{array}\right],$$

$$T^{\left(2\right)}=\left[T_{ij}^{\left(2\right)}\right]=\left[\begin{array}{cccccccc} 1 & 0.45 & 0.18 & 0.027 & 0.0067 & 0.004 & 0.0022 & 0.00067\\ 1 & 0.6 & 0.15 & 0.0825 & 0.0289 & 0.013 & 0.0071 & 0.0014\\ 1 & 0.15 & 0.0412 & 0.0124 & 0.0025 & 0.000495 & 0.00017 & 0.00004 \end{array}\right],$$

$$T^{\left(3\right)}=\left[T_{ij}^{\left(3\right)}\right]=\left[\begin{array}{cccccccc} 1 & 0.3 & 0.09 & 0.0405 & 0.0081 & 0.0036 & 0.0013 & 0.00064\\ 1 & 0.425 & 0.255 & 0.0637 & 0.0351 & 0.0193 & 0.0063 & 0.0022\\ 1 & 0.6 & 0.27 & 0.1215 & 0.0243 & 0.0085 & 0.00085 & 0.00021 \end{array}\right].$$

**Step I-3.** Utilize the IVDHFPWA operator to aggregate the ${\bar{\tilde{r}}}_{ij}^{\left(k\right)}(i=1,2,3;j=1,2,\ldots ,8;$ k=1,2,3), then we get the decision results ${\tilde{r}}_{i}^{\left(k\right)}$ corresponding to the alternative $x_{i}$, where

${\tilde{r}}_{1}^{\left(1\right)}$ = ({[0.5769, 0.6787], [0.5775, 0.6792]}, {[0.1463, 0.2534], [0.1463, 0.2534]});
${\tilde{r}}_{2}^{\left(1\right)}$ = ({[0.3911, 0.6344], [0.3912, 0.6345]}, {[0.213, 0.3389], [0.2155, 0.3423]});
${\tilde{r}}_{3}^{\left(1\right)}$ = ({[0.5459, 0.7367], [0.546, 0.7368], [0.5715, 0.7566], [0.5716, 0.7567]}, {[0.1093, 0.2119], [0.1111, 0.2146], [0.1331, 0.2378], [0.1352, 0.2408]});
${\tilde{r}}_{1}^{\left(2\right)}$ = ({[0.2251, 0.4168], [0.2379, 0.4281], [0.2779, 0.4616], [0.2898, 0.4721]}, {[0.2755, 0.3777]});
${\tilde{r}}_{2}^{\left(2\right)}$ = ({[0.5812, 0.721]}, {[0.1471, 0.2566]});
${\tilde{r}}_{3}^{\left(2\right)}$ = ({[0.3811, 0.4874], [0.3812, 0.4874]}, {[0.2864, 0.4012], [0.3121, 0.4125], [0.3635, 0.4827], [0.3962, 0.4963]});
${\tilde{r}}_{1}^{\left(3\right)}$ = ({[0.1888, 0.3805], [0.2301, 0.4223]}, {[0.307, 0.4273], [0.3071, 0.4274], [0.3105, 0.4315], [0.3106, 0.4315], [0.4373, 0.5658], [0.4375, 0.5659], [0.4423, 0.5713], [0.4425, 0.5714]});
${\tilde{r}}_{2}^{\left(3\right)}$ = ({[0.5009, 0.6697], [0.5036, 0.6723], [0.6012, 0.7183], [0.6034, 0.7205]}, {[0.1171, 0.2778], [0.1173, 0.278], [0.1184, 0.2786], [0.1186, 0.2788]});
${\tilde{r}}_{3}^{\left(3\right)}$ = ({[0.6372, 0.7407]}, {[0.1115, 0.2429], [0.1223, 0.2564]}).

**Step I-4.** Aggregate all the individual overall decision values ${\tilde{r}}_{i}^{\left(k\right)}(k=1,2,3)$ into the group overall decision values ${\tilde{r}}_{i}(i=1,2,3)$ by IVDHFWA operator, where λ=[0.3,0.2,0.5]. Take the ${\tilde{r}}_{1}$ as an example, we have

${\tilde{r}}_{1}$ = ({[0.2619, 0.4457], [0.2809, 0.4647], [0.2644, 0.4479], [0.2833, 0.4668], [0.2722, 0.4545], [0.291, 0.4732], [0.2747, 0.4566], [0.2934, 0.4753], [0.2626, 0.4463], [0.2816, 0.4653], [0.265, 0.4485], [0.284, 0.4674], [0.2729, 0.4551], [0.2917, 0.4738], [0.2753, 0.4572], [0.294, 0.4759]}, {[0.2402, 0.3563], [0.2403, 0.3563], [0.2416, 0.358], [0.2416, 0.358], [0.2867, 0.41], [0.2868, 0.41], [0.2883, 0.412], [0.2884, 0.412], [0.2402, 0.3563], [0.2403, 0.3563], [0.2416, 0.358], [0.2417, 0.3581], [0.2867, 0.41], [0.2868, 0.41], [0.2884, 0.412], [0.2884, 0.412]}).

**Step I-5.** Obtain the score values of $s({\tilde{r}}_{i})$ according to the overall decision values ${\tilde{r}}_{i}(i=1,2,3)$, we have

$$s({\tilde{r}}_{1})=0.0453,s({\tilde{r}}_{2})=0.381,s({\tilde{r}}_{3})=0.4292.$$

Then we can get the ranking order of emergency response solutions as

$$x_{3}≻x_{2}≻x_{1}.$$

Therefore, Approach I indicates that solution $x_{3}$ is the most desirable solution. 

#### 6.1.2. Decision Making Steps of Approach II

**Step II-1.** See Step I-1. 

**Step II-2.** Combine each prioritized individual interval-valued dual hesitant fuzzy decision matrix ${\bar{\tilde{R}}}^{\left(k\right)}={\left({\bar{\tilde{r}}}_{ij}^{\left(k\right)}\right)}_{3\times 8}={\left({\bar{\tilde{h}}}_{ij}^{\left(k\right)},{\bar{\tilde{g}}}_{ij}^{\left(k\right)}\right)}_{3\times 8}(k=1,2,3)$ into the prioritized group interval-valued dual hesitant fuzzy decision matrix $\bar{\tilde{R}}={\left({\bar{\tilde{r}}}_{ij}\right)}_{3\times 8}={\left({\bar{\tilde{h}}}_{ij},{\bar{\tilde{g}}}_{ij}\right)}_{3\times 8}$, as shown in Table 10. 

**Step II-3.** Calculate the $e({\bar{\tilde{r}}}_{ij})$ by Equations (4) and (5), then determine the prioritized levels $T_{ij}(i=1,2,3;j=1,2,\ldots ,8)$ according to Equations (23) and (24) in the prioritized group interval-valued dual hesitant fuzzy decision matrix, where

$$T=\left[T_{ij}\right]=\left[\begin{array}{cccccccc} 1 & 0.4125 & 0.1547 & 0.0348 & 0.0106 & 0.0056 & 0.0028 & 0.0008\\ 1 & 0.4625 & 0.1696 & 0.0608 & 0.0243 & 0.0101 & 0.0034 & 0.0011\\ 1 & 0.3917 & 0.1423 & 0.0605 & 0.0136 & 0.0052 & 0.0017 & 0.0004 \end{array}\right].$$

**Step II-4.** With help of the Matlab^®^ software, utilize the IVDHFPWA operator to aggregate the ${\bar{\tilde{r}}}_{ij}$, we can get the decision results ${\tilde{r}}_{i}$ corresponding to each alternative $x_{i}(i=1,2,3)$. 

**Step II-5.** Calculate the $s({\tilde{r}}_{i})$ of the alternative ${\tilde{r}}_{i}$, where

$$s({\tilde{r}}_{1})=0.052,\;s({\tilde{r}}_{2})=0.3659,\;s({\tilde{r}}_{3})=0.3456.$$

Then we obtain ranking order of the three alternatives according to the $s({\tilde{r}}_{i})$:

$$x_{2}≻x_{3}≻x_{1}.$$

That is, Approach II yields that solution $x_{2}$ is the most desirable solution. 

As can be seen from the ranking order results, both Approach I and Approach II are capable of identifying solution $x_{1}$ as the worst option, but the ranking relations between solution $x_{2}$ and solution $x_{3}$ differs. In order to further show the impact of different weights of decision makers on ranking order of solutions, we also ran the Approach I by setting the weights of decision makers as λ=[0.1,0.4,0.5]. For clarity, the ranking results which were derived from three configurations have been collected in Table 11. As can be observed, when we set λ=[0.1,0.4,0.5] in Approach I, solution $x_{2}$ remained as the same most desirable one. Clearly, different relative importance among decision makers do affect the ranking results of assessing alternatives. One may choose Approach I or Approach II to resolving practical ERSE problems with or without weighting vector of decision makers, respectively. 

### 6.2. Comparative Study with Classic MCGDM Methodologies without Considering Prioritization Relations among Assessing Criteria

To further demonstrate the validity our proposed prioritized operators-based approaches in differentiating response solutions, in this section, we firstly design another two approaches of classic MCGDM methodologies without considering prioritization relations among assessment criteria, that is, $C_{\sigma (j)}=C_{j}$; then, based on the derived ranking results, comparative analyses are further presented to show effectiveness. 

#### 6.2.1. TOPSIS-Based MCGDM Approach

In viewing of the wide acceptance and robustness of TOPSIS methodology, we here design a TOPSIS-based MCGDM approach as shown in following Approach III to resolve the same example adopted in Section 6.1, but without considering prioritization relations among assessing criteria.

**Approach** **III.** **Interval-valued dual hesitant fuzzy MCGDM based on TOPSIS without considering prioritization relations among assessing criteria**

**Step III-1.** Combine each IVDHF decision matrix ${\tilde{R}}^{\left(k\right)}={\left({\tilde{r}}_{ij}^{\left(k\right)}\right)}_{n\times m}={\left({\tilde{h}}_{ij}^{\left(k\right)},{\tilde{g}}_{ij}^{\left(k\right)}\right)}_{n\times m}$ (k=1,2,…,t) listed in Table 4, Table 5 and Table 6 into the group decision matrix $\tilde{R}={\left({\tilde{r}}_{ij}\right)}_{n\times m}={\left({\tilde{h}}_{ij},{\tilde{g}}_{ij}\right)}_{n\times m}$, where ${\tilde{h}}_{ij}=\cup_{{\tilde{\mu }}_{ij}\in {\tilde{h}}_{ij}}\left\{{\tilde{\mu }}_{ij}\right\}$ and ${\tilde{g}}_{ij}=\cup_{{\tilde{\nu }}_{ij}\in {\tilde{g}}_{ij}}\left\{{\tilde{\nu }}_{ij}\right\}$. 

**Step III-2.** Calculate the separating measure from positive and negative ideal solutions. 

Determine positive ideal solution (PIS) $r^{+}=(r_{1}^{+},r_{2}^{+},\ldots ,r_{j}^{+},\ldots ,r_{m}^{+})$ and negative ideal solution (NIS) $r^{-}=(r_{1}^{-},r_{2}^{-},\ldots ,r_{j}^{-},\ldots ,r_{m}^{-})$, where $r_{j}^{+}=(\{1\},\{0\})$, $r_{j}^{-}=(\{0\},\{1\})$. 

Then calculate the separating measure from the PIS and NIS for each alternative according to the distance measure introduced in Equation (5), we have

(28) $$d\left({\tilde{r}}_{ij},{\tilde{r}}_{j}^{+}\right)=\frac{1}{2l_{1}}\sum_{j=1}^{l_{1}}\left(\left|\mu_{ij}^{L}-1\right|+\left|\mu_{ij}^{U}-1\right|\right)+\frac{1}{2l_{2}}\sum_{j=1}^{l_{2}}\left(\left|\nu_{ij}^{L}-0\right|+\left|\nu_{ij}^{U}-0\right|\right),$$

(29) $$d\left({\tilde{r}}_{ij},{\tilde{r}}_{j}^{-}\right)=\frac{1}{2l_{1}}\sum_{j=1}^{l_{1}}\left(\left|\mu_{ij}^{L}-0\right|+\left|\mu_{ij}^{U}-0\right|\right)+\frac{1}{2l_{2}}\sum_{j=1}^{l_{2}}\left(\left|\nu_{ij}^{L}-1\right|+\left|\nu_{ij}^{U}-1\right|\right),$$

and we obtain:

$$d_{i}^{+}=\sum_{j=1}^{m}d({\tilde{r}}_{ij},{\tilde{r}}_{j}^{+})\;\mathrm{and}\;d_{i}^{-}=\sum_{j=1}^{m}d({\tilde{r}}_{ij},{\tilde{r}}_{j}^{-}).$$

**Step III-3.** Calculate the relative closeness to the ideal solution by:

(30) $$c_{i}=\frac{d_{i}^{-}}{d_{i}^{-}+d_{i}^{+}}.$$

**Step III-4.** Obtain ranking order of emergency response solutions according to descending order of $c_{i}$. 

Now, we apply the above Approach III to resolve the decision making problem adopted in Section 6.1. Calculation steps are detailed as follows. 

**Step III-1.** Combine each IVDHF decision matrix ${\tilde{R}}^{\left(k\right)}={\left({\tilde{r}}_{ij}^{\left(k\right)}\right)}_{n\times m}={\left({\tilde{h}}_{ij}^{\left(k\right)},{\tilde{g}}_{ij}^{\left(k\right)}\right)}_{n\times m}$ (k=1,2,…,t) listed in Table 4, Table 5 and Table 6 into the group decision matrix $\tilde{R}={\left({\tilde{r}}_{ij}\right)}_{n\times m}={\left({\tilde{h}}_{ij},{\tilde{g}}_{ij}\right)}_{n\times m}$, as shown in following Table 12. 

**Step III-2.** Calculate the separating measure from positive and negative ideal solutions. Based on group decision matrix $\tilde{R}$, we have

$$d_{1}^{+}=6.7917,\;d_{1}^{-}=9.2083;\;d_{2}^{+}=6.2875,\;d_{2}^{-}=9.7125;\;d_{3}^{+}=7.245,\;d_{3}^{-}=8.755.$$

**Step III-3.** Calculate the relative closeness to the ideal solution, then we have

$$c_{1}=0.5818,\;c_{2}=0.607,\;c_{3}=0.5472.$$

**Step III-4.** Rank the emergency alternatives according to descending order of $c_{i}$. Thus the ranking order can be derived as

$$x_{2}≻x_{1}≻x_{3}.$$

#### 6.2.2. MCGDM Approach Based on Simple Averaging Operator

In this subsection, we follow the aggregation operator-based methodology to devise another MCGDM approach based on a simplified version of IVDHFWA operator [31] in Equation (6) to treat all decision makers with equal importance, which is introduced in the following Approach IV.

**Approach** **IV.** **MCGDM based on Interval-valued dual hesitant fuzzy averaging operator considering prioritization relations among assessing criteria**

**Step IV-1.** Utilize the interval- valued dual hesitant fuzzy averaging (*IVDHFA*) operator, which actually is a special case of IVDHFWA operator [31] in Equation (6) with equal input weights, to aggregate the ${\tilde{r}}_{ij}^{\left(k\right)}(i=1,2,\ldots ,n;j=1,2,\ldots ,m;k=1,2,\ldots ,t)$, then we get the decision results ${\tilde{r}}_{i}^{\left(k\right)}$ corresponding to the alternative $x_{i}$, where

$${\tilde{r}}_{i}^{\left(k\right)}=IVDHFA({\tilde{r}}_{i1}^{\left(k\right)},{\tilde{r}}_{i2}^{\left(k\right)},\ldots ,{\tilde{r}}_{ij}^{\left(k\right)},\ldots ,{\tilde{r}}_{im}^{\left(k\right)})=\;\cup_{[\mu_{ij}^{Lk},\mu_{ij}^{Uk}]\in {\tilde{h}}_{ij}^{\left(k\right)},[\nu_{ij}^{Lk},\nu_{ij}^{Uk}]\in {\tilde{g}}_{ij}^{\left(k\right)}}\left(\left\{\left[1-\prod_{j=1}^{m}{\left(1-\mu_{ij}^{Lk}\right)}^{\frac{1}{m}},1-\prod_{j=1}^{m}{\left(1-\mu_{ij}^{Uk}\right)}^{\frac{1}{m}}\right]\right\},\left\{\left[\prod_{j=1}^{m}{\left(\nu_{ij}^{Lk}\right)}^{\frac{1}{m}},\prod_{j=1}^{m}{\left(\nu_{ij}^{Uk}\right)}^{\frac{1}{m}}\right]\right\}\right).$$

**Step IV-2.** Aggregate all individual overall decision values ${\tilde{r}}_{i}^{\left(k\right)}(k=1,2,\ldots ,t)$ into the group overall decision values ${\tilde{r}}_{i}(i=1,2,\ldots ,n)$ by *IVDHFA* operator, where

$${\tilde{r}}_{i}=IVDHFA({\tilde{r}}_{i}^{\left(1\right)},{\tilde{r}}_{i}^{\left(2\right)},\ldots ,{\tilde{r}}_{i}^{\left(k\right)},\ldots ,{\tilde{r}}_{i}^{\left(t\right)})=\;\cup_{[\mu_{i}^{Lk},\mu_{i}^{Uk}]\in {\tilde{h}}_{i}^{\left(k\right)},[\nu_{i}^{Lk},\nu_{i}^{Uk}]\in {\tilde{g}}_{i}^{\left(k\right)}}\left(\left\{\left[1-\prod_{k=1}^{t}{\left(1-\mu_{i}^{Lk}\right)}^{\frac{1}{t}},1-\prod_{k=1}^{t}{\left(1-\mu_{i}^{Uk}\right)}^{\frac{1}{t}}\right]\right\},\left\{\left[\prod_{k=1}^{t}{\left(\nu_{i}^{Lk}\right)}^{\frac{1}{t}},\prod_{k=1}^{t}{\left(\nu_{i}^{Uk}\right)}^{\frac{1}{t}}\right]\right\}\right).$$

**Step IV-3.** Calculate score values of overall decision values. By use of Equation (2), we get score values $s({\tilde{r}}_{i})$ for the overall decision values ${\tilde{r}}_{i}$, respectively.

**Step IV-4.** Rank alternative emergency response solutions according to descending order of score values $s({\tilde{r}}_{i})$ (i=1,2,…,n). 

Subsequently, we also apply above Approach IV to resolve the same problem in Section 6.1, but not considering prioritization relations among assessing criteria. Decision steps are detailed as follows. 

**Step IV-1.** Utilize the *IVDHFA* operator to aggregate ${\tilde{r}}_{ij}^{\left(k\right)}(i=1,2,3;j=1,2,\ldots ,8;$ k=1,2,3), into ${\tilde{r}}_{i}^{\left(k\right)}$ corresponding to alternative $x_{i}$, then we have

${\tilde{r}}_{1}^{\left(1\right)}$ = ({[0.477, 0.6203], [0.4846, 0.6266]}, {[0.2057, 0.3193], [0.2163, 0.331]});
${\tilde{r}}_{2}^{\left(1\right)}$ = ({[0.3627, 0.5063], [0.3748, 0.5174]}, {[0.2707, 0.396], [0.2769, 0.4037]});
${\tilde{r}}_{3}^{\left(1\right)}$ = ({[0.443, 0.588], [0.4537, 0.5973], [0.4775, 0.6222], [0.4875, 0.6307]}, {[0.2128, 0.3337], [0.2206, 0.3431], [0.232, 0.351], [0.2405, 0.3609]});
${\tilde{r}}_{1}^{\left(2\right)}$ = ({[0.4473, 0.6222], [0.4554, 0.6285], [0.4578, 0.6307], [0.4658, 0.6369]}, {[0.2213, 0.3386]});
${\tilde{r}}_{2}^{\left(2\right)}$ = ({[0.4909, 0.6626]}, {[0.1755, 0.3105]});
${\tilde{r}}_{3}^{\left(2\right)}$ = ({[0.3614, 0.5003], [0.3736, 0.5116]}, {[0.2783, 0.4027], [0.3035, 0.4141], [0.2885, 0.4141], [0.3146, 0.4258]});
${\tilde{r}}_{1}^{\left(3\right)}$ = ({[0.4191, 0.5738], [0.4371, 0.5914]}, {[0.2013, 0.3219], [0.2196, 0.3386], [0.2146, 0.3386], [0.234, 0.3562], [0.206, 0.3281], [0.2246, 0.3452], [0.2196, 0.3452], [0.2394, 0.3631]});
${\tilde{r}}_{2}^{\left(3\right)}$ = ({[0.4949, 0.669], [0.5128, 0.6854], [0.5199, 0.6807], [0.5369, 0.6965]}, {[0.1251, 0.2671], [0.1435, 0.2769], [0.1488, 0.2847], [0.1707, 0.2952]});
${\tilde{r}}_{3}^{\left(3\right)}$ = ({[0.5228, 0.5834]}, {[0.2426, 0.3871], [0.2646, 0.4072]});

**Step IV-2.** Aggregate all the individual overall decision values ${\tilde{r}}_{i}^{\left(k\right)}(k=1,2,3)$ into group overall decision values ${\tilde{r}}_{i}(i=1,2,3)$ by *IVDHFA* operator. Taking ${\tilde{r}}_{1}$ as example, we have

${\tilde{r}}_{1}$ = ({[0.4483, 0.6061], [0.4541, 0.6116], [0.451, 0.6083], [0.4567, 0.6137], [0.4518, 0.609], [0.4576, 0.6145], [0.4545, 0.6112], [0.4602, 0.6166], [0.451, 0.6083], [0.4567, 0.6137], [0.4537, 0.6104], [0.4594, 0.6159], [0.4545, 0.6112], [0.4602, 0.6166], [0.4572, 0.6134], [0.4629, 0.6188]}, {[0.2093, 0.3265], [0.2154, 0.332], [0.2138, 0.332], [0.22, 0.3377], [0.2109, 0.3286], [0.217, 0.3342], [0.2154, 0.3342], [0.2217, 0.3399], [0.2128, 0.3304], [0.2191, 0.336], [0.2174, 0.336], [0.2238, 0.3418], [0.2145, 0.3325], [0.2207, 0.3382], [0.2191, 0.3382], [0.2255, 0.344]}).

**Step IV-3.** Obtain the score values of $s({\tilde{r}}_{i})$ according to the overall decision values ${\tilde{r}}_{i}(i=1,2,3)$, where

$$s({\tilde{r}}_{1})=0.2578,\;s({\tilde{r}}_{2})=0.2851,\;s({\tilde{r}}_{3})=0.1911.$$

**Step IV-4.** Then according to THE descending order of above $s({\tilde{r}}_{i})$ (i=1,2,3), we get the ranking order of emergency response solutions as

$$x_{2}≻x_{1}≻x_{3}.$$

#### 6.2.3. Comparative Analysis

Among the above-developed four approaches, Approach I and Approach II are capable of including criteria prioritization in their decision-making processes, while Approach III and Approach IV are only applicable to general problems without any need to consider prioritization relations among assessment criteria. 

For more clarity, we have collected all the results that were generated by the four approaches in following Table 13. As can be seen from the ranking results, response solutions $x_{1}$ and $x_{2}$ always keep their ranking relations as $x_{2}≻x_{1}$, but the response solution $x_{3}$ holds various positions among ranking results obtained by the four approaches. Regarding the decision situations without considering criteria prioritization, both Approach III and Approach IV comprehensively identified solution $x_{3}$ as the worst one. However, with respect to other situations where there exist criteria prioritization as ($C_{2}$) ≻ ($C_{5}$) ≻ ($C_{1}$) ≻ ($C_{8}$) ≻ ($C_{4}$) ≻ ($C_{6}$) ≻ ($C_{7}$) ≻ ($C_{3}$), we find that solution $x_{3}$ received better evaluations as listed in Table 4, Table 5 and Table 6 than the other two solutions under criteria $C_{2}$, $C_{5}$ and $C_{1}$, which were determined with significantly higher relative importance than other criteria. As a result, Approach I and Approach II, that are capable of exploiting prioritization relations among assessing criteria, both promote response solution $x_{3}$ in their output ranking orders. 

As observed from the above comparative analysis, the proposed Approach I and Approach II manage to reflect important group opinion on criteria prioritization in decision making processes, thus are capable of deriving ranking results rationally when addressing our targeted special type of ERSE problems. 

### 6.3. Comparative Study with Other Methods under a Regressed Decision-Making Scenario

Thus far, to our best knowledge, there are no other MCGDM approaches that address the same practical questions as targeted in this paper, that is, multiple criteria group decision-making problems in which prioritization relations exist among assessment criteria and decision information takes the form of interval-valued dual hesitant fuzzy elements. 

Regarding most related parallel research under dual hesitant fuzzy environments, Ren and Wei [57] developed another methodology based on dice similarity measure to cope with the multiple criteria decision making problems with prioritization relations among criteria, but their method is only applicable to the specific single-person decision-making scenarios with dual hesitant fuzzy information. 

Therefore, to further inspect the flexibility and effectiveness of our proposed approaches, we here firstly regress them to the same special case: single-person dual hesitant fuzzy multiple criteria decision making, then apply to the problem investigated in Ren and Wei [57]. The dual hesitant fuzzy decision matrix given by Ren and Wei [57] is shown in the following Table 14. 

According to the decision matrix shown in Table 14 and the method by Ren and Wei [57], the un-normalized importance weight matrix T and the corresponding normalized importance weight matrix W can be obtained as following

$$T=\left[T_{ij}\right]=\left[\begin{array}{cccc} 1 & 0.6466 & 0.1361 & 0.053\\ 1 & 0.8 & 0.622 & 0.2246\\ 1 & 0.7894 & 0.2968 & 0.203\\ 1 & 0.3648 & 0.2071 & 0.0763\\ 1 & 0.7 & 0.3934 & 0.1312 \end{array}\right]\;\mathrm{and}\;W=\left[w_{ij}\right]=\left[\begin{array}{cccc} 0.5448 & 0.3522 & 0.0742 & 0.0289\\ 0.3778 & 0.3023 & 0.235 & 0.0849\\ 0.4368 & 0.3448 & 0.1297 & 0.0887\\ 0.6054 & 0.2231 & 0.1254 & 0.0462\\ 0.4495 & 0.3147 & 0.1768 & 0.0590 \end{array}\right],$$

based on which, the dice similarity measure [57], correlation coefficient method [83] and the DHFWA operator [84], respectively, have been applied to obtain the ranking orders of all the five solutions, as shown in Table 15. Subsequently, for comparison, we apply our approaches to the same problem.

Firstly, we transform the original decision matrix in Table 14 into interval-valued dual hesitant fuzzy decision matrix, as shown in following Table 16.

Obviously, we can treat the comparative problem as degraded special case and apply our approaches to resolve it. Moreover, please note that, under single-person decision making scenarios, Approach I and Approach II share all the same decision steps. 

Then, according decision steps described in Section 5, we can obtain the unnormalized importance weight matrix $T^{\prime }$ and the corresponding normalized importance weight matrix $W^{\prime }$ as follows:

$$T^{\prime }=\left[T_{i,j}^{\prime }\right]=\left[\begin{array}{cccc} 1 & 0.25 & 0.1375 & 0.0275\\ 1 & 0.6 & 0.3 & 0.165\\ 1 & 0.55 & 0.11 & 0.0385\\ 1 & 0.25 & 0.05 & 0.0125\\ 1 & 0.4 & 0.08 & 0.024 \end{array}\right]\;\mathrm{and}\;W^{\prime }=\left[w_{ij}^{\prime }\right]=\left[\begin{array}{cccc} 0.7067 & 0.1767 & 0.0972 & 0.0194\\ 0.4843 & 0.2906 & 0.1453 & 0.0799\\ 0.5888 & 0.3238 & 0.0648 & 0.0227\\ 0.7619 & 0.1905 & 0.0381 & 0.0095\\ 0.6649 & 0.266 & 0.0532 & 0.016 \end{array}\right],$$

based on which, we get the scores of all five alternatives as: $s(A_{1})$ = 0.1121, $s(A_{2})$ = 0.458, $s(A_{3})$ = 0.3514, $s(A_{4})$ = −0.1675 and $s(A_{5})$ = 0.303, which indicates the ranking result of $A_{2}≻A_{3}≻A_{5}≻A_{1}≻A_{4}$. For more clarity, all the comparative ranking results have been collected in Table 15. 

As can be seen from Table 15, Method I and Method II output the exact same ranking order of all five alternatives, i.e., $A_{2}≻A_{5}≻A_{3}≻A_{1}≻A_{4}$; Method III and our approaches yield the exact same ranking order $A_{2}≻A_{3}≻A_{5}≻A_{1}≻A_{4}$. Although the ranking relation between alternative $A_{3}$ and alternative $A_{5}$ differs above results, the four methods unanimously recognized the best solution $A_{2}$ and the worst solution $A_{4}$, respectively. Therefore, it can be observed that our proposed approaches attain effectiveness and adaptability. 

## 7. Conclusions

In the case of potential developmental risks, emergency response solutions evaluation (ERSE) has become an indispensable regular activity of emergency departments in different levels of modern sustainable governance. 

Focusing on a special type of practical ERSE problems, where decision makers hesitate about their uncertain assessments and prioritization relation exists among assessment criteria, we have developed two effective MCGDM approaches by employing interval-valued dual hesitant fuzzy set (IVDHFS) to help elicit hesitant fuzzy decision information more flexibly and comprehensively. Differing from classic hesitant fuzzy distance measure-based methodologies, we firstly defined a fuzzy entropy measure for IVDHFS so as to avoid information distortion that could be caused by subjective supplementing mechanism. To rationally exploit decision information embedded in prioritization relation among assessing criteria, we have integrated Yager’s prioritized average aggregation operators with the defined IVDHFS fuzzy entropy to develop two prioritized average aggregation operators. On the strength of the above components, we have further constructed two MCGDM approaches to tackle complex ERSE problems with or without known weights of decision makers. Case study and comparative studies have verified the effectiveness and flexibility of our proposed approaches. As can be seen, the methodology presented in this paper is of obvious practical significance in helping emergency departments construct rational models of their decision support systems, thereby well maintaining response repositories in case of evolving emergency events. 

Due to the fast pace of economic development in many countries, new issues will be brought about to ERSE activities by operational demands in emergency management, such as, the requirement of appropriate decision making frameworks that accommodates large group of heterogeneous decision makers (stakeholders), even in settings of distributed networks. Therefore, future research directions should be conducted along following lines: firstly, cross-entropy measure and correlational coefficient could be introduced to derive programming models in order to determine unknown weighting information objectively; secondly, large-group uncertain decision making methods with heterogeneous decision information should be developed in-depth to accommodate involved stakeholders; thirdly, novel group decision making frameworks in the scenarios of distributed networks also should be paid more attention. In addition, comprehensive evaluation of practices in collaborative emergency response management deserves further efforts to identify critical factors so that governments can build more effectiveness and resilience into emergency response solutions. 

## Figures and Tables

**Table 1:** Activity phase-oriented criteria for ERSE.

Phases	Criteria	Meanings of the Criteria
Before-activity	Response time to start emergency response solution ($C_{1}$) [1,14]	Projected least time interval between identified alert of emergency event and start-up of emergency response solution, which generally comprises of activities for collection of first-hand information event, expert team call-up, etc.
	Reasonable organizational structure and clear awareness of responsibilities ($C_{2}$) [1,14]	Rationality in configuration of organizational structure and clearness of corresponding tasks and responsibilities.
	Economic cost ($C_{3}$) [70,71]	Budget for estimated expenses of carrying out the emergency response solution.
During-activity	Operability of the response solution ($C_{4}$) [1,66]	Operational effectiveness in execution of the response solution, such as aspects on complexity of task division, appropriate inclusion of modern emergency equipment, etc.
	Monitoring and forecasting potential hazards ($C_{5}$) [1,14,70,71]	Capacity of utilizing scientific approaches, such as information system and decision support system, to monitor influencing factors and thereby identifying or forecasting potential hazards.
	Reconstruction ability ($C_{6}$) [1,14]	Response solution’s capacity in recovery of public infrastructure, public utilities or housing in event areas.
After-activity	Social impact ($C_{7}$) [72]	Capacity to appropriately cope with derivative social risks caused by the emergency event or emergency response solution, such as public panic, mass violent events in areas of electricity outage due to emergent response actions.
	Environmental impact ($C_{8}$) [73,74]	Estimated consequences that could be caused be response solutions on the local environment of event spots.

**Table 2:** Example emergency events and their derived prioritization phenomena among the assessing criteria in Table 1.

Event Title	Event Description	Deduced Prioritizations among Assessing Criteria
1. Chemical spills	(i) Located nearby a city drinking water reservoir. (ii) Will seriously contaminate the reservoir, thus cause problems in water supply. (iii) Needs collaboration between many city departments and water supply needs quick recovery to avoid public panic. (iv) Chemical fluid materials spilled will cause environmental damage to rivers along water conveyance to the reservoir. (v) Response solution cost can be covered by municipal public finance.	($C_{2}$) ≻ ($C_{5}$) ≻ ($C_{1}$) ≻ ($C_{8}$) ≻ ($C_{4}$) ≻ ($C_{6}$) ≻ ($C_{7}$) ≻ ($C_{3}$)
2. Hazardous materials tank truck crash	(i) Located on a freeway bridge of typically heavy traffic in mountain area and there is the main river under the bridge. (ii) Truck drivers were not injured. (iii) Scarcely any residences nearby. (iv) A large amount of corrosive fluid materials in both trucks are leaking and about to come out quickly. (v) Fluid materials exposed will irreversibly damage the river and local environment.	($C_{2}$) ≻ ($C_{5}$) ≻ ($C_{1}$) ≻ ($C_{4}$) ≻ ($C_{6}$) ≻ ($C_{8}$) ≻ ($C_{7}$) ≻ ($C_{3}$)
3. Hazardous materials tank truck crash	(i) Located on an intersection of two highways in a sandstorm desert area where no residences are nearby. (ii) Truck drivers were injured. (iii) A large amount of highly corrosive fluid materials in both trucks are leaking. (iv) Accident trucks destroyed a critical sand control dam.	($C_{1}$) ≻ ($C_{4}$) ≻ ($C_{2}$) ≻ ($C_{5}$) ≻ ($C_{6}$) ≻ ($C_{3}$) ≻ ($C_{7}$) ≻ ($C_{8}$)

**Table 3:** Decision matrix given by the *k*-th decision maker.

$R^{k}$ (*k* = 1, …, *t*)		*Criterion with Its Prioritization Level*			
		$C_{1}^{u}$	$C_{2}^{u}$	…	$C_{m}^{u}$
*Solutions*	$x_{1}$	$r_{11}^{k}$	$r_{12}^{k}$	…	$r_{1m}^{k}$
	$x_{2}$	$r_{21}^{k}$	$r_{22}^{k}$	…	$r_{2m}^{k}$
	⋮	⋮	⋮	⋮	⋮
	$x_{n}$	$r_{n1}^{k}$	$r_{n2}^{k}$	…	$r_{nm}^{k}$

**Table 4:** The IVDHF decision matrix ${\tilde{R}}^{\left(1\right)}$ provided by decision maker $E_{1}$.

	$C_{1}$	$C_{2}$	$C_{3}$	$C_{4}$
$x_{1}$	({[0.4, 0.5]}, {[0.4, 0.5]})	({[0.6, 0.7]}, {[0.1, 0.2]})	({[0.1, 0.4]}, {[0.2, 0.3], [0.3, 0.4]})	({[0.6, 0.8]}, {[0.1, 0.2]})
$x_{2}$	({[0.2, 0.3]}, {[0.5, 0.6], [0.6, 0.7]})	({[0.4, 0.7]}, {[0.2, 0.3]})	({[0.5, 0.6]}, {[0.1, 0.2]})	({[0.4, 0.5]}, {[0.3, 0.4]})
$x_{3}$	({[0.5, 0.6], [0.7, 0.8]}, {[0.1, 0.2]})	({[0.6, 0.8]}, {[0.1, 0.2]})	({[0.3, 0.4], [0.4, 0.5]}, {[0.4, 0.5]})	({[0.7, 0.8]}, {[0.1, 0.2]})
	$C_{5}$	$C_{6}$	$C_{7}$	$C_{8}$
$x_{1}$	({[0.6, 0.7]}, {[0.2, 0.3]})	({[0.7, 0.8]}, {[0.1, 0.2]})	({[0.4, 0.5]}, {[0.4, 0.5]})	({[0.1, 0.2], [0.2, 0.3]}, {[0.5, 0.6]})
$x_{2}$	({[0.4, 0.5]}, {[0.2, 0.4]})	({[0.3, 0.4], [0.4, 0.5]}, {[0.4, 0.5]})	({[0.1, 0.3]}, {[0.6, 0.7]})	({[0.5, 0.6]}, {[0.2, 0.3]})
$x_{3}$	({[0.5, 0.7]}, {[0.1, 0.2], [0.2, 0.3]})	({[0.1, 0.2]}, {[0.7, 0.8]})	({[0.3, 0.4]}, {[0.5, 0.6]})	({[0.3, 0.4]}, {[0.3, 0.4], [0.4, 0.5]})

**Table 5:** The IVDHF decision matrix ${\tilde{R}}^{\left(2\right)}$ provided by decision maker $E_{2}$.

	$C_{1}$	$C_{2}$	$C_{3}$	$C_{4}$
$x_{1}$	({[0.3, 0.4], [0.4, 0.5]}, {[0.4, 0.5]})	({[0.1, 0.2], [0.2, 0.3]}, {[0.3, 0.4]})	({[0.6, 0.7]}, {[0.2, 0.3]})	({[0.7, 0.8]}, {[0.1, 0.2]})
$x_{2}$	({[0.6, 0.8]}, {[0.1, 0.2]})	({[0.7, 0.8]}, {[0.1, 0.2]})	({[0.2, 0.3]}, {[0.5, 0.6]})	({[0.6, 0.7]}, {[0.1, 0.3]})
$x_{3}$	({[0.2, 0.5]}, {[0.3, 0.4]})	({[0.4, 0.5]}, {[0.3, 0.4], [0.4, 0.5]})	({[0.7, 0.8]}, {[0.1, 0.2]})	({[0.3, 0.4]}, {[0.5, 0.6]})
	$C_{5}$	$C_{6}$	$C_{7}$	$C_{8}$
$x_{1}$	({[0.4, 0.7]}, {[0.2, 0.3]})	({[0.6, 0.8]}, {[0.1, 0.2]})	({[0.2, 0.4]}, {[0.4, 0.6]})	({[0.4, 0.6]}, {[0.3, 0.4]})
$x_{2}$	({[0.3, 0.5]}, {[0.3, 0.4]})	({[0.6, 0.8]}, {[0.1, 0.2]})	({[0.3, 0.5]}, {[0.3, 0.5]})	({[0.4, 0.6]}, {[0.2, 0.3]})
$x_{3}$	({[0.3, 0.4]}, {[0.2, 0.4], [0.4, 0.5]})	({[0.3, 0.4], [0.4, 0.5]}, {[0.2, 0.3]})	({[0.2, 0.4]}, {[0.5, 0.6]})	({[0.3, 0.4]}, {[0.4, 0.5]})

**Table 6:** The IVDHF decision matrix ${\tilde{R}}^{\left(3\right)}$ provided by decision maker $E_{3}$.

	$C_{1}$	$C_{2}$	$C_{3}$	$C_{4}$
$x_{1}$	({[0.1, 0.2] }, {[0.5, 0.6], [0.6, 0.7]})	({[0.2, 0.4]}, {[0.3, 0.4], [0.5, 0.6]})	({[0.6, 0.7]}, {[0.1, 0.2], [0.2, 0.3]})	({[0.5, 0.7]}, {[0.1, 0.2]})
$x_{2}$	({[0.4, 0.6]}, {[0.3, 0.4]})	({[0.4, 0.6], [0.6, 0.7]}, {[0.1, 0.3]})	({[0.3, 0.5]}, {[0.1, 0.3], [0.4, 0.5]})	({[0.6, 0.7], [0.7, 0.8]}, {[0.1, 0.2]})
$x_{3}$	({[0.6, 0.7]}, {[0.1, 0.2], [0.2, 0.3]})	({[0.7, 0.8]}, {[0.1, 0.2]})	({[0.5, 0.6]}, {[0.3, 0.4]})	({[0.2, 0.3]}, {[0.5, 0.7]})
	$C_{5}$	$C_{6}$	$C_{7}$	$C_{8}$
$x_{1}$	({[0.1, 0.3], [0.3, 0.5]}, {[0.3, 0.5]})	({[0.5, 0.7]}, {[0.2, 0.3]})	({[0.6, 0.7]}, {[0.1, 0.2]})	({[0.5, 0.6]}, {[0.3, 0.4]})
$x_{2}$	({[0.7, 0.8]}, {[0.1, 0.2]})	({[0.3, 0.5]}, {[0.1, 0.3], [0.3, 0.4]})	({[0.5, 0.7]}, {[0.2, 0.3]})	({[0.6, 0.8]}, {[0.1, 0.2]})
$x_{3}$	({[0.6, 0.7]}, {[0.1, 0.3]})	({[0.4, 0.5]}, {[0.4, 0.5]})	({[0.2, 0.4]}, {[0.5, 0.6]})	({[0.3, 0.4]}, {[0.4, 0.5]})

**Table 7:** The prioritized individual IVDHF decision matrix ${\bar{\tilde{R}}}^{\left(1\right)}$.

	$C_{\sigma (1)}$	$C_{\sigma (2)}$	$C_{\sigma (3)}$	$C_{\sigma (4)}$
$x_{1}$	({[0.6, 0.7]}, {[0.1, 0.2]})	({[0.6, 0.7]}, {[0.2, 0.3]})	({[0.4, 0.5]}, {[0.4, 0.5]})	({[0.1, 0.2], [0.2, 0.3]}, {[0.5, 0.6]})
$x_{2}$	({[0.4, 0.7]}, {[0.2, 0.3]})	({[0.4, 0.5]}, {[0.2, 0.4]})	({[0.2, 0.3]}, {[0.5, 0.6], [0.6, 0.7]})	({[0.5, 0.6]}, {[0.2, 0.3]})
$x_{3}$	({[0.6, 0.8]}, {[0.1, 0.2]})	({[0.5, 0.7]}, {[0.1, 0.2], [0.2, 0.3]})	({[0.5, 0.6], [0.7, 0.8]}, {[0.1, 0.2]})	({[0.3, 0.4]}, {[0.3, 0.4], [0.4, 0.5]})
	$C_{\sigma (5)}$	$C_{\sigma (6)}$	$C_{\sigma (7)}$	$C_{\sigma (8)}$
$x_{1}$	({[0.6, 0.8]}, {[0.1, 0.2]})	({[0.7, 0.8]}, {[0.1, 0.2]})	({[0.4, 0.5]}, {[0.4, 0.5]})	({[0.1, 0.4]}, {[0.2, 0.3], [0.3, 0.4]})
$x_{2}$	({[0.4, 0.5]}, {[0.3, 0.4]})	({[0.3, 0.4], [0.4, 0.5]}, {[0.4, 0.5]})	({[0.1, 0.3]}, {[0.6, 0.7]})	({[0.5, 0.6]}, {[0.1, 0.2]})
$x_{3}$	({[0.7, 0.8]}, {[0.1, 0.2]})	({[0.1, 0.2]}, {[0.7, 0.8]})	({[0.3, 0.4]}, {[0.5, 0.6]})	({[0.3, 0.4], [0.4, 0.5]}, {[0.4, 0.5]})

**Table 8:** The prioritized individual IVDHF decision matrix ${\bar{\tilde{R}}}^{\left(2\right)}$.

	$C_{\sigma (1)}$	$C_{\sigma (2)}$	$C_{\sigma (3)}$	$C_{\sigma (4)}$
$x_{1}$	({[0.1, 0.2], [0.2, 0.3]}, {[0.3, 0.4]})	({[0.4, 0.7]}, {[0.2, 0.3]})	({[0.3, 0.4], [0.4, 0.5]}, {[0.4, 0.5]})	({[0.4, 0.6]}, {[0.3, 0.4]})
$x_{2}$	({[0.7, 0.8]}, {[0.1, 0.2]})	({[0.3, 0.5]}, {[0.3, 0.4]})	({[0.6, 0.8]}, {[0.1, 0.2]})	({[0.4, 0.6]}, {[0.2, 0.3]})
$x_{3}$	({[0.4, 0.5]}, {[0.3, 0.4], [0.4, 0.5]})	({[0.3, 0.4]}, {[0.2, 0.4], [0.4, 0.5]})	({[0.2, 0.5]}, {[0.3, 0.4]})	({[0.3, 0.4]}, {[0.4, 0.5]})
	$C_{\sigma (5)}$	$C_{\sigma (6)}$	$C_{\sigma (7)}$	$C_{\sigma (8)}$
$x_{1}$	({[0.7, 0.8]}, {[0.1, 0.2]})	({[0.6, 0.8]}, {[0.1, 0.2]})	({[0.2, 0.4]}, {[0.4, 0.6]})	({[0.6, 0.7]}, {[0.2, 0.3]})
$x_{2}$	({[0.6, 0.7]}, {[0.1, 0.3]})	({[0.6, 0.8]}, {[0.1, 0.2]})	({[0.3, 0.5]}, {[0.3, 0.5]})	({[0.2, 0.3]}, {[0.5, 0.6]})
$x_{3}$	({[0.3, 0.4]}, {[0.5, 0.6]})	({[0.3, 0.4], [0.4, 0.5]}, {[0.2, 0.3]})	({[0.2, 0.4]}, {[0.5, 0.6]})	({[0.7, 0.8]}, {[0.1, 0.2]})

**Table 9:** The prioritized individual IVDHF decision matrix ${\bar{\tilde{R}}}^{\left(3\right)}$.

	$C_{\sigma (1)}$	$C_{\sigma (2)}$	$C_{\sigma (3)}$	$C_{\sigma (4)}$
$x_{1}$	({[0.2, 0.4]}, {[0.3, 0.4], [0.5, 0.6]})	({[0.1, 0.3], [0.3, 0.5]}, {[0.3, 0.5]})	({[0.1, 0.2] }, {[0.5, 0.6], [0.6, 0.7]})	({[0.5, 0.6]}, {[0.3, 0.4]})
$x_{2}$	({[0.4, 0.6], [0.6, 0.7]}, {[0.1, 0.3]})	({[0.7, 0.8]}, {[0.1, 0.2]})	({[0.4, 0.6]}, {[0.3, 0.4]})	({[0.6, 0.8]}, {[0.1, 0.2]})
$x_{3}$	({[0.7, 0.8]}, {[0.1, 0.2]})	({[0.6, 0.7]}, {[0.1, 0.3]})	({[0.6, 0.7]}, {[0.1, 0.2], [0.2, 0.3]})	({[0.3, 0.4]}, {[0.4, 0.5]})
	$C_{\sigma (5)}$	$C_{\sigma (6)}$	$C_{\sigma (7)}$	$C_{\sigma (8)}$
$x_{1}$	({[0.5, 0.7]}, {[0.1, 0.2]})	({[0.5, 0.7]}, {[0.2, 0.3]})	({[0.6, 0.7]}, {[0.1, 0.2]})	({[0.6, 0.7]}, {[0.1, 0.2], [0.2, 0.3]})
$x_{2}$	({[0.6, 0.7], [0.7, 0.8]}, {[0.1, 0.2]})	({[0.3, 0.5]}, {[0.1, 0.3], [0.3, 0.4]})	({[0.5, 0.7]}, {[0.2, 0.3]})	({[0.3, 0.5]}, {[0.1, 0.3], [0.4, 0.5]})
$x_{3}$	({[0.2, 0.3]}, {[0.5, 0.7]})	({[0.4, 0.5]}, {[0.4, 0.5]})	({[0.2, 0.4]}, {[0.5, 0.6]})	({[0.5, 0.6]}, {[0.3, 0.4]})

**Table 10:** The prioritized group IVDHF matrix $\bar{\tilde{R}}$.

	$C_{\sigma (1)}$	$C_{\sigma (2)}$	$C_{\sigma (3)}$	$C_{\sigma (4)}$
$x_{1}$	({[0.1, 0.2], [0.2, 0.3], [0.2, 0.4], [0.6, 0.7]}, {[0.1, 0.2], [0.3, 0.4], [0.3, 0.4], [0.5, 0.6]})	({[0.1, 0.3], [0.3, 0.5], [0.4, 0.7], [0.6, 0.7]}, {[0.2, 0.3], [0.2, 0.3], [0.3, 0.5]})	({[0.1, 0.2], [0.3, 0.4], [0.4, 0.5], [0.4, 0.5]}, {[0.4, 0.5], [0.4, 0.5], [0.5, 0.6], [0.6, 0.7]})	({[0.1, 0.2], [0.2, 0.3], [0.4, 0.6], [0.5, 0.6]}, {[0.3, 0.4], [0.3, 0.4], [0.5, 0.6]})
$x_{2}$	({[0.4, 0.6], [0.4, 0.7], [0.6, 0.7], [0.7, 0.8]}, {[0.1, 0.2], [0.1, 0.3], [0.2, 0.3]})	({[0.3, 0.5], [0.4, 0.5], [0.7, 0.8]}, {[0.1, 0, 2], [0.2, 0.4], [0.3, 0.4]})	({[0.2, 0.3], [0.4, 0.6], [0.6, 0.8]}, {[0.1, 0.2], [0.3, 0.4], [0.5, 0.6], [0.6, 0.7]})	({[0.4, 0.6], [0.5, 0.6], [0.6, 0.8]}, {[0.1, 0.2], [0.2, 0.3], [0.2, 0.3]})
$x_{3}$	({[0.4, 0.5], [0.6, 0.8], [0.7, 0.8]}, {[0.1, 0.2], [0.1, 0.2], [0.3, 0.4], [0.4, 0.5]})	({[0.3, 0.4], [0.5, 0.7], [0.6, 0.7]}, {[0.1, 0.2], [0.1, 0.3], [0.2, 0.3], [0.2, 0.4], [0.4, 0.5]})	({[0.2, 0.5], [0.5, 0.6], [0.6, 0.7], [0.7, 0.8]}, {[0.1, 0.2], [0.1, 0.2], [0.2, 0.3], [0.3, 0.4]})	({[0.3, 0.4], [0.3, 0.4], [0.3, 0.4]}, {[0.3, 0.4], [0.4, 0.5], [0.4, 0.5], [0.4, 0.5]})
	$C_{\sigma (5)}$	$C_{\sigma (6)}$	$C_{\sigma (7)}$	$C_{\sigma (8)}$
$x_{1}$	({[0.5, 0.7], [0.6, 0.8], [0.7, 0.8]}, {[0.1, 0.2], [0.1, 0.2], [0.1, 0.2]})	({[0.5, 0.7], [0.6, 0.8], [0.7, 0.8]}, {[0.1, 0.2], [0.1, 0.2], [0.2, 0.3]})	({[0.2, 0.4], [0.4, 0.5], [0.6, 0.7]}, {[0.1, 0.2], [0.4, 0.5], [0.4, 0.6]})	({[0.1, 0.4], [0.6, 0.7], [0.6, 0.7]}, {[0.1, 0.2], [0.2, 0.3], [0.2, 0.3], [0.2, 0.3], [0.3, 0.4]})
$x_{2}$	({[0.4, 0.5], [0.6, 0.7], [0.6, 0.7], [0.7, 0.8]}, {[0.1, 0.2], [0.1, 0.3], [0.3, 0.4]})	({[0.3, 0.4], [0.3, 0.5], [0.4, 0.5], [0.6, 0.8]}, {[0.1, 0.2], [0.1, 0.3], [0.3, 0.4], [0.4, 0.5]})	({[0.1, 0.3], [0.3, 0.5], [0.5, 0.7]}, {[0.2, 0.3], [0.3, 0.5], [0.6, 0.7]})	({[0.2, 0.3], [0.3, 0.5], [0.5, 0.6]}, {[0.1, 0.2], [0.1, 0.3], [0.4, 0.5], [0.5, 0.6]})
$x_{3}$	({[0.2, 0.3], [0.3, 0.4], [0.7, 0.8]}, {[0.1, 0.2], [0.5, 0.6], [0.5, 0.7]})	({[0.1, 0.2], [0.3, 0.4], [0.4, 0.5], [0.4, 0.5]}, {[0.2, 0.3], [0.4, 0.5], [0.7, 0.8]})	({[0.2, 0.4], [0.2, 0.4], [0.3, 0.4]}, {[0.5, 0.6], [0.5, 0.6], [0.5, 0.6]})	({[0.3, 0.4], [0.4, 0.5], [0.5, 0.6], [0.7, 0.8]}, {[0.1, 0.2], [0.3, 0.4], [0.4, 0.5]})

**Table 11:** Ranking orders obtained by Approach I and Approach II.

Methods	Ranking Orders Obtained	Scores of Alternatives
**Approach I** (λ=[0.3,0.2,0.5])	$x_{3}≻x_{2}≻x_{1}$	$s({\tilde{r}}_{1})$ = 0.0453, $s({\tilde{r}}_{2})$ = 0.381, $s({\tilde{r}}_{3})$ = 0.4292.
**Approach I** (λ=[0.1,0.4,0.5])	$x_{2}≻x_{3}≻x_{1}$	$s({\tilde{r}}_{1})$ = 0.0070, $s({\tilde{r}}_{2})$ = 0.4201, $s({\tilde{r}}_{3})$ = 0.3554.
**Approach II**	$x_{2}≻x_{3}≻x_{1}$	$s({\tilde{r}}_{1})$ = 0.052, $s({\tilde{r}}_{2})$ = 0.3659, $s({\tilde{r}}_{3})$ = 0.3456.

**Table 12:** The group IVDHF decision matrix $\tilde{R}$.

	$C_{\sigma (1)}$	$C_{\sigma (2)}$	$C_{\sigma (3)}$	$C_{\sigma (4)}$
$x_{1}$	({[0.1, 0.2], [0.3, 0.4], [0.4, 0.5], [0.4, 0.5]}, {[0.4, 0.5], [0.4, 0.5], [0.5, 0.6], [0.6, 0.7]})	({[0.1, 0.2], [0.2, 0.3], [0.2, 0.4], [0.6, 0.7]}, {[0.1, 0.2], [0.3, 0.4], [0.3, 0.4], [0.5, 0.6]})	({[0.1, 0.4], [0.6, 0.7], [0.6, 0.7]}, {[0.1, 0.2], [0.2, 0.3], [0.2, 0.3], [0.2, 0.3], [0.3, 0.4]})	({[0.5, 0.7], [0.6, 0.8], [0.7, 0.8]}, {[0.1, 0.2], [0.1, 0.2], [0.1, 0.2]})
$x_{2}$	({[0.2, 0.3], [0.4, 0.6], [0.6, 0.8]}, {[0.1, 0.2], [0.3, 0.4], [0.5, 0.6], [0.6, 0.7]})	({[0.4, 0.6], [0.4, 0.7], [0.6, 0.7], [0.7, 0.8]}, {[0.1, 0.2], [0.1, 0.3], [0.2, 0.3]})	({[0.2, 0.3], [0.3, 0.5], [0.5, 0.6]}, {[0.1, 0.2], [0.1, 0.3], [0.4, 0.5], [0.5, 0.6]})	({[0.4, 0.5], [0.6, 0.7], [0.6, 0.7], [0.7, 0.8]}, {[0.1, 0.2], [0.1, 0.3], [0.3, 0.4]})
$x_{3}$	({[0.2, 0.5], [0.5, 0.6], [0.6, 0.7], [0.7, 0.8]}, {[0.1, 0.2], [0.1, 0.2], [0.2, 0.3], [0.3, 0.4]})	({[0.4, 0.5], [0.6, 0.8], [0.7, 0.8]}, {[0.1, 0.2], [0.1, 0.2], [0.3, 0.4], [0.4, 0.5]})	({[0.3, 0.4], [0.4, 0.5], [0.5, 0.6], [0.7, 0.8]}, {[0.1, 0.2], [0.3, 0.4], [0.4, 0.5]})	({[0.2, 0.3], [0.3, 0.4], [0.7, 0.8]}, {[0.1, 0.2], [0.5, 0.6], [0.5, 0.7]})
	$C_{\sigma (5)}$	$C_{\sigma (6)}$	$C_{\sigma (7)}$	$C_{\sigma (8)}$
$x_{1}$	({[0.1, 0.3], [0.3, 0.5], [0.4, 0.7], [0.6, 0.7]}, {[0.2, 0.3], [0.2, 0.3], [0.3, 0.5]})	({[0.5, 0.7], [0.6, 0.8], [0.7, 0.8]}, {[0.1, 0.2], [0.1, 0.2], [0.2, 0.3]})	({[0.2, 0.4], [0.4, 0.5], [0.6, 0.7]}, {[0.1, 0.2], [0.4, 0.5], [0.4, 0.6]})	({[0.1, 0.2], [0.2, 0.3], [0.4, 0.6], [0.5, 0.6]}, {[0.3, 0.4], [0.3, 0.4], [0.5, 0.6]})
$x_{2}$	({[0.3, 0.5], [0.4, 0.5], [0.7, 0.8]}, {[0.1, 0, 2], [0.2, 0.4], [0.3, 0.4]})	({[0.3, 0.4], [0.3, 0.5], [0.4, 0.5], [0.6, 0.8]}, {[0.1, 0.2], [0.1, 0.3], [0.3, 0.4], [0.4, 0.5]})	({[0.1, 0.3], [0.3, 0.5], [0.5, 0.7]}, {[0.2, 0.3], [0.3, 0.5], [0.6, 0.7]})	({[0.4, 0.6], [0.5, 0.6], [0.6, 0.8]}, {[0.1, 0.2], [0.2, 0.3], [0.2, 0.3]})
$x_{3}$	({[0.3, 0.4], [0.5, 0.7], [0.6, 0.7]}, {[0.1, 0.2], [0.1, 0.3], [0.2, 0.3], [0.2, 0.4], [0.4, 0.5]})	({[0.1, 0.2], [0.3, 0.4], [0.4, 0.5], [0.4, 0.5]}, {[0.2, 0.3], [0.4, 0.5], [0.7, 0.8]})	({[0.2, 0.4], [0.2, 0.4], [0.3, 0.4]}, {[0.5, 0.6], [0.5, 0.6], [0.5, 0.6]})	({[0.3, 0.4], [0.3, 0.4], [0.3, 0.4]}, {[0.3, 0.4], [0.4, 0.5], [0.4, 0.5], [0.4, 0.5]})

**Table 13:** Ranking orders obtained by Approaches I–IV.

Methods	Obtained Ranking Orders	Scores of Solutions
**Approach I** (considering weights for decision makers and prioritization among criteria)	$x_{3}≻x_{2}≻x_{1}$	$s({\tilde{r}}_{1})$ = 0.0453, $s({\tilde{r}}_{2})$ = 0.381, $s({\tilde{r}}_{3})$ = 0.4292.
**Approach II** (considering prioritization among criteria)	$x_{2}≻x_{3}≻x_{1}$	$s({\tilde{r}}_{1})$ = 0.052, $s({\tilde{r}}_{2})$ = 0.3659, $s({\tilde{r}}_{3})$ = 0.3456.
**Approach III** (not considering prioritization among criteria)	$x_{2}≻x_{1}≻x_{3}$	$c_{1}$ = 0.5818, $c_{2}$ = 0.607, $c_{3}$ = 0.5472.
**Approach IV** (not considering prioritization among criteria)	$x_{2}≻x_{1}≻x_{3}$	$s({\tilde{r}}_{1})$ = 0.2578, $s({\tilde{r}}_{2})$ = 0.2851, $s({\tilde{r}}_{3})$ = 0.1911.

**Table 14:** Dual hesitant fuzzy decision matrix of the comparative example adopted in Ren and Wei [57].

	$G_{1}$	$G_{2}$	$G_{3}$	$G_{4}$
$A_{1}$	{(0.5, 0.6), (0.3)}	{(0.2), (0.7, 0.8)}	{(0.3, 0.4), (0.5, 0.6)}	{(0.5, 0.6, 0.7), (0.3)}
$A_{2}$	{(0.8), (0.2)}	{(0.6, 0.7, 0.8), (0.2)}	{(0.1, 0.2), (0.3)}	{(0.2), (0.6, 0.7, 0.8)}
$A_{3}$	{(0.7, 0.8), (0.2)}	{(0.2, 0.3, 0.4), (0.5)}	{(0.4, 0.5), (0.2)}	{(0.2, 0.4), (0.5, 0.6)}
$A_{4}$	{(0.3, 0.4), (0.6)}	{(0.4, 0.5), (0.3, 0.4)}	{(0.3, 0.4), (0.6)}	{(0.4, 0.5), (0.5)}
$A_{5}$	{(0.7), (0.3)}	{(0.4, 0.5), (0.3, 0.4)}	{(0.3), (0.5, 0.6, 0.7)}	{(0.5), ( 0.4, 0.5)}

**Table 15:** Ranking results obtained by comparative methods *.

Methods	Ranking Orders Obtained	Scores of Alternatives
**Method I**: Score function [57] + dice similarity measure [57]	$A_{2}≻A_{5}≻A_{3}≻A_{1}≻A_{4}$	$PD(A^{*},A_{1})$ = 0.7156; $PD(A^{*},A_{2})$ = 0.8477; $PD(A^{*},A_{3})$ = 0.8287; $PD(A^{*},A_{4})$ = 0.7004; $PD(A^{*},A_{5})$ = 0.8459.
**Method II:** Score function [57] + correlation coefficient method [83]	$A_{2}≻A_{5}≻A_{3}≻A_{1}≻A_{4}$	$\rho_{DHFS}(A^{*},A_{1})$ = 0.6008; $\rho_{DHFS}(A^{*},A_{2})$ = 0.8073; $\rho_{DHFS}(A^{*},A_{3})$ = 0.7680; $\rho_{DHFS}(A^{*},A_{4})$ = 0.5625; $\rho_{DHFS}(A^{*},A_{5})$ = 0.7841.
**Method III:** Score function [57] + DHFWA operator [84]	$A_{2}≻A_{3}≻A_{5}≻A_{1}≻A_{4}$	$s(A_{1})$ = 0.0039; $s(A_{2})$ = 0.4012; $s(A_{3})$ = 0.2707; $s(A_{4})$ = −0.1468; $s(A_{5})$ = 0.2026.
**Method IV:** Our Approaches	$A_{2}≻A_{3}≻A_{5}≻A_{1}≻A_{4}$	$s(A_{1})$ = 0.1121; $s(A_{2})$ = 0.458; $s(A_{3})$ = 0.3514; $s(A_{4})$ = −0.1675; $s(A_{5})$ = 0.303.

* We have programmed all the comparative methods by use of Matlab^®^ and verified the results listed.

**Table 16:** Interval-valued Dual hesitant fuzzy decision matrix of the comparative example adopted in Ren and Wei [57].

	$G_{1}$	$G_{2}$	$G_{3}$	$G_{4}$
$A_{1}$	{([0.5, 0.5], [0.6, 0.6]), ([0.3, 0.3])}	{([0.2, 0.2]), ([0.7, 0.7], [0.8, 0.8])}	{([0.3, 0.3], [0.4, 0.4]), ([0.5, 0.5], [0.6, 0.6])}	{([0.5, 0.5], [0.6, 0.6], [0.7, 0.7]), ([0.3, 0.3])}
$A_{2}$	{([0.8, 0.8]), ([0.2, 0.2])}	{([0.6, 0.6], [0.7, 0.7], [0.8, 0.8]), ([0.2, 0.2])}	{([0.1, 0.1], [0.2, 0.2]), ([0.3, 0.3])}	{([0.2, 0.2]), ([0.6, 0.6], [0.7, 0.7], [0.8, 0.8])}
$A_{3}$	{([0.7, 0.7], [0.8, 0.8]), ([0.2, 0.2])}	{([0.2, 0.2], [0.3, 0.3], [0.4, 0.4]), ([0.5, 0.5])}	{([0.4, 0.4], [0.5, 0.5]), ([0.2, 0.2])}	{([0.2, 0.2], [0.4, 0.4]), ([0.5, 0.5], [0.6, 0.6])}
$A_{4}$	{([0.3, 0.3], [0.4, 0.4]), ([0.6, 0.6])}	{([0.4, 0.4], [0.5, 0.5]), ([0.3, 0.3], [0.4, 0.4])}	{([0.3, 0.3], [0.4, 0.4]), ([0.6, 0.6])}	{([0.4, 0.4], [0.5, 0.5]), ([0.5, 0.5])}
$A_{5}$	{([0.7, 0.7]), ([0.3, 0.3])}	{([0.4, 0.4], [0.5, 0.5]), ([0.3, 0.3], [0.4, 0.4])}	{([0.3, 0.3]), ([0.5, 0.5], [0.6, 0.6], [0.7, 0.7])}	{([0.5, 0.5]), ([0.4, 0.4], [0.5, 0.5])}

## References

[1] Ju Y., Wang A., You T. (2015). Emergency alternative evaluation and selection based on ANP, DEMATEL, and TL-TOPSIS. Nat. Hazards.

[2] Wu W., Peng Y. (2016). Extension of grey relational analysis for facilitating group consensus to oil spill emergency management. Ann. Oper. Res..

[3] OECD (2003). OECD Guiding Principles for Chemical Accident Prevention, Preparedness and Response.

[4] US-EPA Hazardous Waste Operations and Emergency Response: General Information and Comparison. https://www.epa.gov/laws-regulations/regulations.

[5] European-Commission Directive 2012/18/EU Of The European Parliament And Of The Council. http://eur-lex.europa.eu/LexUriServ/LexUriServ.do?uri=OJ:L:2012:197:0001:0037:EN:PDF.

[6] Duan W., He B. (2015). Emergency response system for pollution accidents in chemical industrial parks, China. Int. J. Environ. Res. Public Health.

[7] Shao C., Yang J., Tian X., Ju M., Huang L. (2013). Integrated environmental risk assessment and whole-process management system in chemical industry parks. Int. J. Environ. Res. Public Health.

[8] Wan C., Zhang D., Yan X., Yang Z. (2017). A novel model for the quantitative evaluation of green port development—A case study of major ports in China. Transp. Res. Part D.

[9] Zhang J., Hegde G., Shang J., Qi X. (2016). Evaluating emergency response solutions for sustainable community development by using fuzzy multi-criteria group decision making approaches: IVDHF-TOPSIS and IVDHF-VIKOR. Sustainability.

[10] Busi E., Maranghi S., Corsi L., Basosi R. (2016). Environmental sustainability evaluation of innovative self-cleaning textiles. J. Clean. Prod..

[11] Köhler A.R., Som C. (2014). Risk preventative innovation strategies for emerging technologies the cases of nano-textiles and smart textiles. Technovation.

[12] Whiteley C.E., Boguski T., Erickson L., Anthony J.L., Green R. (2009). Emergency preparation and green engineering: Augmenting the environmental knowledge and assessment tool. Environ. Prog. Sustain..

[13] Fogli D., Guida G. (2013). Knowledge-centered design of decision support systems for emergency management. Decis. Support Syst..

[14] Ju Y., Wang A. (2012). Emergency alternative evaluation under group decision makers: A method of incorporating DS/AHP with extended TOPSIS. Expert Syst. Appl..

[15] Ju Y., Wang A., Liu X. (2012). Evaluating emergency response capacity by fuzzy AHP and 2-tuple fuzzy linguistic approach. Expert Syst. Appl..

[16] Ju Y., Yang S. (2015). A new method for multiple attribute group decision-making with intuitionistic trapezoid fuzzy linguistic information. Soft Comput..

[17] Wang L., Zhang Z.-X., Wang Y.-M. (2015). A prospect theory-based interval dynamic reference point method for emergency decision making. Expert Syst. Appl..

[18] Mardani A., Jusoh A., Zavadskas E.K. (2015). Fuzzy multiple criteria decision-making techniques and applications—Two decades review from 1994 to 2014. Expert Syst. Appl..

[19] Hashemi S.S., Hajiagha S.H.R., Zavadskas E.K., Mahdiraji H.A. (2016). Multicriteria group decision making with ELECTRE III method based on interval-valued intuitionistic fuzzy information. Appl. Math. Model..

[20] Zavadskas E.K., Kaklauskas A., Sarka V. (1994). The new method of multicriteria complex proportional assessment of projects. Technol. Econ. Dev. Econ..

[21] Yazdani M., Jahan A., Zavadskas E.K. (2017). Analysis in material selection: Influence of normalization tools on Copras-G. Econ. Comput. Econ. Cybern. Stud. Res..

[22] Zavadskas E.K., Turskis Z. (2010). A new additive ratio assessment (ARAS) method in multicriteria decision-making. Ukio Technol. Ekon. Vystym..

[23] Stanujkic D., Zavadskas E.K., Karabasevic D., Turskis Z., Keršulienė V. (2017). New group decision-making ARCAS approach based on the integration of the SWARA and the ARAS methods adapted for negotiations. J. Bus. Econ. Manag..

[24] Brauers W.K.M., Zavadskas E.K. (2010). Project management by multimoora as an instrument for transition economies. Ukio Technol. Ekon. Vystym..

[25] Zavadskas E.K., Bausys R., Juodagalviene B., Garnyte-Sapranaviciene I. (2017). Model for residential house element and material selection by neutrosophic MULTIMOORA method. Eng. Appl. Artif. Intell..

[26] Stanujkic D., Zavadskas E.K., Smarandache F., Brauers W.K.M., Karabasevic D. (2017). A neutrosophic extension of the MULTIMOORA method. Informatica.

[27] Torra V., Narukawa Y. On hesitant fuzzy sets and decision. Proceedings of the 18th IEEE International Conference on Fuzzy Systems.

[28] Torra V. (2010). Hesitant fuzzy sets. Int. J. Intell. Syst..

[29] Zhu B., Xu Z.S., Xia M.M. (2012). Dual hesitant fuzzy sets. J. Appl. Math..

[30] Farhadinia B. (2014). Correlation for dual hesitant fuzzy sets and dual interval-valued hesitant fuzzy sets. Int. J. Intell. Syst..

[31] Ju Y.B., Liu X.Y., Yang S.H. (2014). Interval-valued dual hesitant fuzzy aggregation operators and their applications to multiple attribute decision making. J. Intell. Fuzzy Syst..

[32] Xu Z.S., Xia M.M. (2011). Distance and similarity measures for hesitant fuzzy sets. Inf. Sci..

[33] Su Z., Xu Z., Liu H., Liu S. (2015). Distance and similarity measures for dual hesitant fuzzy sets and their applications in pattern recognition. J. Intell. Fuzzy Syst..

[34] Zadeh L.A. (1968). Probability measures of Fuzzy events. J. Math. Anal. Appl..

[35] Burillo P., Bustince H. (1996). Entropy on intuitionistic fuzzy sets and on interval-valued fuzzy sets. Fuzzy Sets Syst..

[36] Ye J. (2010). Fuzzy decision-making method based on the weighted correlation coefficient under intuitionistic fuzzy environment. Eur. J. Oper. Res..

[37] Ye J. (2010). Multicriteria fuzzy decision-making method using entropy weights-based correlation coefficients of interval-valued intuitionistic fuzzy sets. Appl. Math. Model..

[38] Qi X., Liang C., Zhang J. (2015). Generalized cross-entropy based group decision making with unknown expert and attribute weights under interval-valued intuitionistic fuzzy environment. Comput. Ind. Eng..

[39] Tian Z.-P., Zhang H.-Y., Wang J., Wang J.-Q., Chen X.-H. (2016). Multi-criteria decision-making method based on a cross-entropy with interval neutrosophic sets. Int. J. Syst. Sci..

[40] Xu Z.S., Xia M.M. (2012). Hesitant fuzzy entropy and cross-entropy and their use in multi-attribute decision making. Int. J. Intell. Syst..

[41] Ye J. (2016). Cross-entropy of dual hesitant fuzzy sets for multiple attribute decision-making. Int. J. Decis. Support Syst. Technol..

[42] Xie K., Chen G., Wu Q., Liu Y., Wang P. (2011). Research on the group decision-making about emergency event based on network technology. Inf. Technol. Manag..

[43] Cao H., Li T., Li S., Fan T. (2016). An integrated emergency response model for toxic gas release accidents based on cellular automata. Ann. Oper. Res..

[44] Shi S., Cao J., Feng L., Liang W., Zhang L. (2014). Construction of a technique plan repository and evaluation system based on AHP group decision-making for emergency treatment and disposal in chemical pollution accidents. J. Hazard. Mater..

[45] Liu J., Guo L., Jiang J., Hao L., Liu R., Wang P. (2015). Evaluation and selection of emergency treatment technology based on dynamic fuzzy GRA method for chemical contingency spills. J. Hazard. Mater..

[46] Yager R.R. (2008). Prioritized aggregation operators. Int. J. Approx. Reason..

[47] Yager R.R. (2009). Prioritized OWA aggregation. Fuzzy Optim. Decis. Mak..

[48] Yu D., Wu Y., Lu T. (2012). Interval-valued intuitionistic fuzzy prioritized operators and their application in group decision making. Knowl.-Based Syst..

[49] Yu X.H., Xu Z.S. (2013). Prioritized intuitionistic fuzzy aggregation operators. Inf. Fusion.

[50] Yu D.J. (2013). Prioritized information fusion method for triangular intuitionistic fuzzy set and its application to teaching quality evaluation. Int. J. Intell. Syst..

[51] Zhao Q.Y., Chen H.Y., Zhou L.G., Tao Z.F., Liu X. (2015). The properties of fuzzy number intuitionistic fuzzy prioritized operators and their applications to multi-criteria group decision making. J. Intell. Fuzzy Syst..

[52] Peng D.H., Wang T.D., Gao C.Y., Wang H. (2013). Multigranular uncertain linguistic prioritized aggregation operators and their application to multiple criteria group decision making. J. Appl. Math..

[53] Chen L., Xu Z. (2015). A new prioritized multi-criteria outranking method: The prioritized Promethee. J. Intell. Fuzzy Syst..

[54] Wei G.W. (2012). Hesitant fuzzy prioritized operators and their application to multiple attribute decision making. Knowl.-Based Syst..

[55] Wu J.T., Wang J.Q., Wang J., Zhang H.Y., Chen X.H. (2014). Hesitant fuzzy linguistic multicriteria decision-making method based on generalized prioritized aggregation operator. Sci. World J..

[56] Jin F., Ni Z., Chen H. (2016). Interval-valued hesitant fuzzy Einstein prioritized aggregation operators and their applications to multi-attribute group decision making. Soft Comput..

[57] Ren Z., Wei C. (2017). A multi-attribute decision-making method with prioritization relationship and dual hesitant fuzzy decision information. Int. J. Mach. Learn. Cybern..

[58] Hu W., Qing Y., Yu M.-H., Qi F. (2008). Grid-based platform for disaster response plan simulation over Internet. Simul. Model. Pract. Theory.

[59] Maldonado E.A., Maitland C.F., Tapia A.H. (2010). Collaborative systems development in disaster relief: The impact of multi-level governance. Inf. Syst. Front..

[60] Koliba C.J., Mills R.M., Zia A. (2011). Accountability in governance networks: An assessment of public, private, and nonprofit emergency management practices following hurricane katrina. Public Adm. Rev..

[61] Kuo M.F., Wang C.Y., Chang Y.Y., Li T.S. (2015). Collaborative Disaster Management: Lessons from Taiwan’s Local Governments.

[62] Noran O. (2014). Collaborative disaster management: An interdisciplinary approach. Comput. Ind..

[63] Kapucu N., Hu Q., Khosa S. (2014). The state of network research in public administration. Adm. Soc..

[64] Guo X., Kapucu N. (2015). Examining collaborative disaster response in China: Network perspectives. Nat. Hazards.

[65] Tseng J.M., Kuo C.Y., Liu M.Y., Shu C.M. (2008). Emergency response plan for boiler explosion with toxic chemical releases at Nan-Kung industrial park in central Taiwan. Process Saf. Environ. Prot..

[66] People’s Republic of China, The-Ministry-of-Civil-Affairs (2016). Emergency Plan for Natural Disaster Rescue.

[67] European Environment Agency (EEA) (2001). Late Lessons from Early Warnings: The Precautionary Principle.

[68] Chen A., Chen N., Li J. (2012). During-incident process assessment in emergency management: Concept and strategy. Saf. Sci..

[69] Phillips B.D., Neal D.M., Webb G. (2012). Introduction to Emergency Management.

[70] People’s Republic of China, The-State-Council (2007). China’s Speical Law for Countermeasures to Emergency Events.

[71] People’s Republic of China, The-Ministry-of-Civil-Affairs (2010). Regulations on Natural Disaster Rscue and Assistance.

[72] Momoh J.A., Zhang Y., Fanara P., Kurban H., Iwarere L.J. (2007). Social impact based contingency screening and ranking. Int. J. Crit. Infrastruct..

[73] Kelly C. (2003). Quick Guide: Rapid Environmental Impact Assessment in Disaster.

[74] Kelly C. (2005). Guidelines for Rapid Environmental Impact Assessment in Disasters.

[75] Zhang J.-L., Qi X.-W. (2012). Induced interval-valued intuitionistic fuzzy hybrid aggregation operators with TOPSIS order-inducing variables. J. Appl. Math..

[76] Kahraman C., Onar S.C., Oztaysi B. (2015). Fuzzy multicriteria decision-making: A literature review. Int. J. Comput. Intell. Syst..

[77] De Luca A., Termini S. (1972). A definition of a nonprobabilistic entropy in the setting of fuzzy sets theory. Inf. Control.

[78] Yager R.R. (1979). On the measure of fuzziness and negation Part I: Membership in the unit interval. Int. J. Gen. Syst..

[79] Szmidt E., Kacprzyk J. (2001). Entropy for intuitionistic fuzzy sets. Fuzzy Sets Syst..

[80] Hung W.-L., Yang M.-S. (2006). Fuzzy entropy on intuitionistic fuzzy sets. Int. J. Intell. Syst..

[81] Xu Z. (2010). A deviation-based approach to intuitionistic fuzzy multiple attribute group decision making. Group Decis. Negot..

[82] Zhao N., Xu Z. Entropy Measures for Dual Hesitant Fuzzy Information. Proceedings of the 2015 Fifth International Conference on Communication Systems and Network Technologies.

[83] Ye J. (2014). Correlation coefficient of dual hesitant fuzzy sets and its application to multiple attribute decision making. Appl. Math. Model..

[84] Wang H.J., Zhao X.F., Wei G.W. (2014). Dual hesitant fuzzy aggregation operators in multiple attribute decision making. J. Intell. Fuzzy Syst..

